# Gauging road safety advances using a hybrid EWM–PROMETHEE II–DBSCAN model with machine learning

**DOI:** 10.3389/fpubh.2024.1413031

**Published:** 2024-08-22

**Authors:** Jialin Li, Faan Chen

**Affiliations:** ^1^College of Arts and Science, Vanderbilt University, Nashville, TN, United States; ^2^School of Engineering and Applied Sciences, Harvard University, Cambridge, MA, United States

**Keywords:** public health, road safety, decision reliability, Southeast Asia, policymaking, machine learning

## Abstract

**Introduction:**

Enhancing road safety conditions alleviates socioeconomic hazards from traffic accidents and promotes public health. Monitoring progress and recalibrating measures are indispensable in this effort. A systematic and scientific decision-making model that can achieve defensible decision outputs with substantial reliability and stability is essential, particularly for road safety system analyses.

**Methods:**

We developed a systematic methodology combining the entropy weight method (EWM), preference ranking organization method for enrichment evaluation (PROMETHEE), and density-based spatial clustering of applications with noise (DBSCAN)—referred to as EWM–PROMETHEE II–DBSCAN—to support road safety monitoring, recalibrating measures, and action planning. Notably, we enhanced DBSCAN with a machine learning algorithm (grid search) to determine the optimal parameters of neighborhood radius and minimum number of points, significantly impacting clustering quality.

**Results:**

In a real case study assessing road safety in Southeast Asia, the multi-level comparisons validate the robustness of the proposed model, demonstrating its effectiveness in road safety decision-making. The integration of a machine learning tool (grid search) with the traditional DBSCAN clustering technique forms a robust framework, improving data analysis in complex environments. This framework addresses DBSCAN’s limitations in nearest neighbor search and parameter selection, yielding more reliable decision outcomes, especially in small sample scenarios. The empirical results provide detailed insights into road safety performance and potential areas for improvement within Southeast Asia.

**Conclusion:**

The proposed methodology offers governmental officials and managers a credible tool for monitoring overall road safety conditions. Furthermore, it enables policymakers and legislators to identify strengths and drawbacks and formulate defensible policies and strategies to optimize regional road safety.

## 1 Introduction

As of 2019, road traffic accidents were the 12th leading cause of death for all age groups (1). This significant loss of life severely impacts human development, exacerbates poverty, negatively affects victims and their families, and has an aggregated effect on the gross domestic product of approximately 3% annually (2). In response to these serious consequences, numerous countries, administrative bodies, and organizations, including the World Health Organization (WHO), have implemented road safety action plans at regional levels. However, the results have been underwhelming. Target 3.6 of the Sustainable Development Goals, which aimed to reduce the number of road traffic deaths and injuries by half by 2020, is currently labeled red (3), indicating no progress or regression from the target. Southeast Asia is particularly challenged by road safety issues, with a road traffic death rate of approximately 20.7 per 100,000 people, significantly higher than the global rate of approximately 18 per 100,000 (4). Given that Southeast Asia houses roughly 8.58% of the global population, monitoring progress and recalibrating interventions are urgently needed to develop a comprehensive understanding of road safety conditions.

Monitoring progress and recalibrating interventions require a scientific, efficient, and reliable approach to form a basis for legislative or policy action. This involves evaluating (i.e., ranking and grouping) alternatives (e.g., countries, states, or jurisdictions) based on various criteria, a process known as multi-criteria decision-making (MCDM). Previous studies in developing such approaches provide a solid foundation for this study (5–10). However, research gaps persist, leading to five research motivations for this study:

- Previous methods, such as data envelopment analysis (DEA)-based approaches (11), a technique for order of preference by similarity to ideal solution–rank-sum ratio (TOPSIS–RSR) (12), and regret theory integrated weighted aggregated sum product assessment (13), have been primarily applied in individual countries or small regions, with few cross-national or large regional applications. They do not sufficiently consider the diversity of countries’ socioeconomic development. As such, there are currently no universally applicable safety performance indicators (SPIs) for gauging road safety conditions within Southeast Asia at a regional level. Thus, a set of SPIs that can provide a holistic picture of road safety is urgently required.
- Many previously developed approaches, such as the distance function approach (14) and DEA for composite indicators (15), stop at the aggregation stage (i.e., ranking the alternatives) without considering grouping, decomposing, and benchmarking, which are vital for detailed and targeted decision-making.
- Most previous models exhibit significant defects, such as model-related sensitivity and outcome uncertainty, leading to issues of decision reliability, particularly with small samples. Therefore, delivering reliable and defensible decisions is a precondition for a qualified MCDM model.
- To date, no approach integrates the multiple steps of the MCDM process—weighting, aggregation, grouping, decomposing, and benchmarking—into a single procedure. A comprehensive MCDM model that encompasses these steps with significant reliability and stability is needed.
- Grouping alternatives is an essential step in MCDM activities, commonly achieved using density-based spatial clustering of applications with noise (DBSCAN). However, DBSCAN has parameter and noise sensitivities, requiring two parameters: the maximum distance between two samples for one to be considered in the neighborhood of the other (𝜖) and the number of points required to form a dense region (𝑚𝑖𝑛𝑃𝑡𝑠). Selecting appropriate values for these parameters can be challenging, and high noise levels can lead to fewer and less meaningful clusters.

To this end, this study constructs a novel MCDM model that combines the entropy weight method (EWM), preference ranking organization method for enrichment evaluation (PROMETHEE), and DBSCAN, referred to as EWM–PROMETHEE II–DBSCAN with grid search. This model aims to provide policymakers and practitioners with a reliable tool for gauging and benchmarking road safety conditions in Southeast Asian countries. The study makes significant contributions to the academic, industrial, and private sectors in three key ways:

(1) The set of SPIs offers a fundamental measurement framework for road safety development in Southeast Asia at the regional level.
(2) The proposed methodology refines the MCDM procedure in road safety and introduces a novel structure for road safety benchmarking, supplementing existing evaluation systems. It particularly addresses constraints of conventional DBSCAN clustering by integrating a machine learning tool (grid search), enhancing clustering performance by identifying optimal parameters for neighborhood radius (*ε*) and 𝑚𝑖𝑛𝑃𝑡𝑠, and resolving challenges associated with closest neighbor search and parameter selection.
(3) It drives political will and government accountability for improved road safety conditions, enabling Southeast Asian countries to conduct periodic self-assessments. This assists governmental officials, legislators, and professionals in identifying weak performance areas and implementing appropriate actions and strategies.

The remainder of the paper is organized as follows. Section 2 reviews the reliability of the MCDM process and road safety conditions in Southeast Asia. Section 3 introduces the set of SPIs and their data sources and details our proposed methodology. Section 4 presents the empirical results of the case study on Southeast Asian countries and the robustness analysis. Section 5 discusses policy and practice implications. Finally, Section 6 concludes with a review of the study’s contributions, limitations, and directions for further research.

## 2 Literature review

### 2.1 Road safety development in Southeast Asia

#### 2.1.1 Factors contributing to traffic accidents

Road safety conditions in Southeast Asia vary significantly among countries due to factors such as infrastructure quality, traffic regulations, enforcement effectiveness, vehicle safety standards, and public awareness, which span the human–vehicle–infrastructure–environment–management system (16).

The human factor (road users) is considered the major contributor to road accidents, including speeding, drunk driving, and the use (or lack thereof) of seat belts and helmets (1, 17). Public awareness campaigns, driver education programs, and initiatives promoting responsible road behavior contribute to improving road safety by raising awareness about risks, promoting safer driving habits, and encouraging compliance with traffic rules (18, 19). Regarding vehicles, adherence to safety standards, including vehicle maintenance, roadworthiness checks, and the use of safety features such as seat belts and airbags, can influence road safety outcomes. The quality of roads and transportation infrastructure varies across regions. While some countries have well-maintained highways and road networks, others may face issues such as potholes, inadequate signage, and lack of pedestrian facilities, impacting overall safety. Many cities in Southeast Asia experience heavy traffic congestion, which can contribute to road safety challenges such as increased accident risks, longer emergency response times, and frustration among drivers (20).

Enforcement of traffic laws and regulations plays a crucial role in ensuring road safety. Countries with effective law enforcement and strict penalties for traffic violations tend to have better safety records compared to those with lax enforcement practices (2, 4, 21). Rapid urbanization and economic development in Southeast Asian countries can lead to increased motorization, higher vehicle ownership rates, and complex traffic dynamics, requiring proactive measures to manage road safety challenges.

While some Southeast Asian countries have significantly improved road safety through infrastructure upgrades, enhanced enforcement, and public awareness campaigns, continuous efforts and investment in comprehensive road safety strategies are needed to reduce accidents, injuries, and fatalities on the region’s roads. In particular, collaboration among Southeast Asian countries through regional organizations such as the Association of Southeast Asian Nations (ASEAN) can facilitate knowledge sharing, exchange of best practices, and coordinated efforts to address common road safety issues.

#### 2.1.2 Road safety measurement activities

Road safety measurement is a critical area for policymakers, practitioners, and stakeholders in Southeast Asia, involving various initiatives and frameworks aimed at monitoring, evaluating, and improving road safety. These activities typically include the collection and analysis of data, the development of safety performance indicators, and the implementation of targeted interventions. The region experiences high rates of road traffic accidents, resulting in significant socioeconomic losses and public health challenges. Over the past decade, significant progress has been made in road safety measurement at both national and regional levels in Southeast Asia.

At the national level, some studies have highlighted the challenges and methodologies involved in setting and achieving road safety targets in rapidly developing countries, such as Cambodia, emphasizing the importance of data and strategic planning in reducing road fatalities (22–24). Other studies have explored the current state of road safety management in Malaysia, focusing on funding and institutional arrangements (25, 26). These studies involved semi-structured interviews with key stakeholders in road safety management, including policymakers, private sector representatives, and academics, to gather insights on the effectiveness and efficiency of road safety initiatives. This comprehensive assessment underscores the importance of a well-coordinated, adequately funded, and institutionally robust road safety management system to reduce road traffic accidents and improve overall road safety in Malaysia. Additionally, one study evaluated the effects of the road safety system approach implemented in Brunei (27).

At the regional level, some studies have assessed road safety performance in Southeast Asian countries, focusing aligning national strategies with the “safe system” vision (28). A road safety assessment index for Southeast Asia was developed using indicators reflecting safe system principles: safer roads, vehicles, and road users. The aforementioned study concluded that countries with higher crash rates are beginning to address safety issues. However, further efforts are needed to implement recommendations from the Decade of Actions on Road Safety 2011–2020. Establishing minimum vehicle safety standards is emphasized as critical for all countries in the region. Other studies have explored the assessment of road safety risk in various Asian countries (29), employing DEA to calculate and rank road safety risk levels and structural equation modeling to analyze the interaction between these risk levels and influencing factors such as financial impact, institutional framework, infrastructure and mobility, legislation and policy, vehicular road users, and trauma management.

Overall, previous studies have significantly contributed to understanding road safety development in Southeast Asia. However, road safety development and performance analysis should be an ongoing diagnostic process conducted regularly. At this critical juncture, serving as a bridge between the past and the next decade, such analysis is particularly valuable for Southeast Asian countries. It helps identify areas of poor performance and potential issues, and more importantly, enables effective oversight of road safety progress. This supports future policy improvements and program development toward achieving the Sustainable Development Goals 2030 target.

### 2.2 SPIs

Road traffic crashes result from a combination of factors involving the components of the road transport system, including road users, vehicles, infrastructure, and their interactions within a broader environment (30). Due to the complexity of road safety, numerous indicators are increasingly suggested for monitoring, evaluating, and comparing road safety status and progress. This approach contrasts with traditional methods, which considered only a few factors, such as safety outcomes measured by fatalities *per capita* or vehicle. Measuring and comparing road safety levels using individual indicators can be misleading, as it does not account for the aggregation of these indicators. This can result in partial or incorrect conclusions as they use different exposure information from various perspectives (31). Recognizing the limitations of traditional approaches that focus solely on mortality or fatality rates, there is a growing global interest in developing composite road safety performance indices. These indices capture a broader road safety perspective, especially over the past two decades (15, 32–38).

Al-Haji (32, 39) proposed the Road Safety Development Index (RSDI), encompassing eight aspects of road safety connected with the human–vehicle–road–environment–regulation system. These dimensions include traffic risk, personal risk, vehicle safety, road conditions, road user behavior, socioeconomic background, road safety organization, and enforcement. Various quantitative indicators were analyzed for their applicability based on available data. To create a composite index (RSDI) by merging SPIs from different domains, three primary methods were employed: simple average, theoretical weights, and principal component analysis (PCA). The outcomes of both methodologies were comparable and facilitated the ranking of nations based on their safety performance.

Wegman et al. (34) proposed a comprehensive and integrated collection of indicators using a composite index known as the SUNflower Index to summarize extensive road safety data. The indicators are classified into three categories: road safety performance (measuring outcomes), implementation performance (measuring processes), and policy performance (assessing the quality of national road safety policies). These indicators are integrated into a policy framework that encompasses the organization and values of a nation, including relevant contextual factors. Statistical techniques such as model-based weighting, PCA, and common factor analysis were used to merge the fundamental indicators into a composite index.

Hermans et al. (40) explored combining road safety information into a performance index using SPIs developed by the European SafetyNet project (41). The SPIs include seven crucial domains vital for enhancing road safety in Europe: alcohol and drug use, speed control, protective systems, implementation of daytime running lights, vehicle standards, road infrastructure, and trauma treatment. The variables were combined into an overall score for 21 European nations using five common weighting approaches: factor analysis, budget allocation, analytic hierarchy process (AHP), DEA, and equal weighting. The rankings of the nations obtained from these methodologies were compared to the standard rankings based on mortality rates *per capita*. The DEA technique most accurately reflected the rankings based on the number of road deaths per million residents. Furthermore, Hermans (35) established a theoretical framework and technique for constructing a performance index. This index serves to condense important information on indicators, with a particular focus on SPIs. A collection of essential and accessible SPIs was established using this framework, and the process for constructing a comprehensive road safety index by integrating all indicator data was explained.

Shen et al. (42) conducted a study on the integration of hierarchically organized SPIs for road safety benchmarking in 28 European nations. This study identified six factors contributing to road safety risks within the human–vehicle–infrastructure system: alcohol consumption, speeding, protection systems, car quality, road conditions, and emergency medical services. An extensive collection of SPIs was developed to provide a detailed understanding of road safety. These indicators are organized in a multilayer hierarchical framework. The study used DEA and its expansions to create a composite road safety performance index, comparing nations’ performance based on several variables. The findings demonstrated a significant correlation between the developed road safety performance indicator and ultimate road safety outcomes.

Bax et al. (33) developed the composite Road Safety Index, which integrates various sets of SPIs to assess road safety performance across three levels: final outcomes (such as injuries and crashes), intermediate outcomes (including factors related to drunk driving, speeding, and car safety), and policy output (safety measures and programs). This index allows for the comparison of road safety across different countries, serving as a model for nations to enhance their road safety efforts. The index considers variances in national structure and culture, resulting in distinct initial groupings of countries. Two separate road safety composite indices were created—one based on road safety result indicators and the other on intermediate outcomes—and these were combined to form the comprehensive Road Safety Composite Index. However, a policy performance index was not developed at that time.

Gitelman et al. (43) created a set of indicators to construct a composite index for evaluating and advancing child road safety at the local level. The framework included six dimensions related to traffic safety: injury, background characteristics, behaviors, attitudes, policy and management, and environment and walkability. An expert team identified key indications within each dimension. These indicators were aggregated using common factor analysis to create a composite index for each domain, except environment and walkability, which was assessed with a single town as a pretest. Safety rankings of towns were visualized using maps based on factor values. Classification trees were generated using weighted factor values and Ward’s clustering approach to identify municipalities with similar characteristics. This study provided government officials and policymakers with child road safety indicators, composite indices, and comparison tools to assess, monitor, and compare the performance of municipalities in child road safety at regional or national levels.

Chen et al. (36) refined and updated Al-Haji’s RSDI framework (32, 39), which integrates a broader range of indicators and was tested for its effectiveness in tracking road safety progress within the ASEAN region. The case study demonstrated the effectiveness of the RSDI framework for regional road safety monitoring, facilitating understanding of road safety trends and supporting coordinated regional efforts to improve safety outcomes.

Tešić et al. (38) explored the methodology for developing a road safety performance index using a limited set of indicators. The study aimed to create a scientifically robust method for monitoring and comparing road safety performance across countries. The proposed methodology standardizes road safety indicators, providing policymakers with a practical tool for comparison. By using a concise set of indicators, the index can be calculated easily while delivering reliable and comparable results. The study highlights the importance of reliable data and appropriate statistical methods to ensure the accuracy and robustness of the road safety performance index.

Shen et al. (15) addressed the challenges and methodologies of international road safety benchmarking. They emphasized the development of a comprehensive set of hierarchically structured SPIs and the application of DEA to create composite indicators that account for data uncertainty and hierarchical structures. The study benchmarks road safety performance across European countries, identifying both leading and underperforming countries. By incorporating country-specific characteristics, the study offers tailored benchmarks and policy recommendations aimed at enhancing road safety management and outcomes using international best practices.

Shbeeb (44) provided an overview of indices used in road safety, highlighting the complexity of road safety issues and the growing interest in benchmarking mechanisms for comparing safety performance. The study assessed road safety performances in 20 selected European Union and African countries using two approaches: one with simple averaging techniques for cross-sectional data and the other with multi-regression analysis of time-series data over six years. The findings suggest that the road safety index can effectively facilitate international comparisons of road safety performances.

Overall, the literature demonstrates that various problem-decomposing methodologies are widely employed in selecting road safety performance indicators. These methodologies include the human–vehicle–infrastructure system, exposure–risk–injury decomposition, the road safety pyramid model, the Haddon matrix–C3-R3 systems approach, or combinations of these approaches.

### 2.3 Reliability of the MCDM procedure

The reliability of the MCDM procedure refers to the consistency and dependability of the decision-making process and outcomes over time (45, 46). It ensures that the MCDM method yields consistent results under similar conditions and that the decisions made are dependable and trustworthy (47). Assessing this reliability can be achieved through various methods, including test–retest reliability, internal consistency, and inter-rater reliability, depending on the specific MCDM technique used (48, 49).

A key aspect of MCDM reliability is the dependability of the criteria and their weights (50). In MCDM, different criteria are often used to evaluate alternatives, and assigning appropriate weights to these criteria is crucial for sound decision-making. To enhance the reliability of MCDM criteria weights, several techniques are commonly employed:

- **Consistency checks:** one primary method for assessing decision reliability in MCDM is through consistency checks. These involve evaluating the consistency of pairwise comparisons or judgments made by decision-makers during the process (51–53). Techniques such as the consistency ratio in the AHP or the consistency index in the analytic network process (ANP) are used to quantify the consistency of judgments. Consistent judgments indicate higher decision reliability.
- **Sensitivity analysis:** sensitivity analysis is another important technique for enhancing decision reliability in MCDM. It involves testing the robustness of decisions by varying criteria weights or input parameters (54, 55). This helps assess the impact of changes in criteria weights on the final decision, ensuring that minor variations in weights do not significantly alter the outcome. By conducting sensitivity analysis, decision-makers can understand how changes in criteria weights or inputs affect the final decision. Robust decisions that remain stable across different scenarios demonstrate higher reliability.
- **Expert validation:** expert validation is crucial for enhancing decision reliability (56). This involves seeking input and feedback from domain experts to validate the criteria weights assigned in the MCDM process. Incorporating expert knowledge and judgment into the decision-making process improves the reliability of the weights. Experts provide insights, validate assumptions, and assess the reliability of outcomes based on their domain experience (57).
- **Risk analysis:** incorporating risk analysis techniques into the MCDM process also contributes to decision reliability. By considering uncertainties, variability, and potential risks associated with different decision alternatives, decision-makers can make more informed choices. Techniques such as probabilistic modeling (58, 59), Monte Carlo simulation (48, 60), and sensitivity to risk factors (54) are valuable for assessing decision reliability in the face of uncertainty.

Aggregation stability under varying conditions is another key aspect (9, 61), encompassing several factors. First, it necessitates producing consistent results despite variations in model parameters and assumptions. This involves assessing how changes in model assumptions impact decision outcomes (62). A stable MCDM process should exhibit reasonable sensitivity to changes without causing drastic shifts in rankings (63, 64). Second, it requires model validation to ensure the MCDM model accurately represents the decision problem and aligns with real-world expectations (65). Validation confirms the stability of the decision-making process. In addition, the coherence and logical consistency of decision preferences throughout the decision-making process are essential (66), as inconsistent preferences can lead to unstable decisions.

Overall, reliability is crucial in MCDM to ensure decisions are reliable, consistent, and robust across various conditions, leading to more informed and effective decision-making.

## 3 Data

### 3.1 Index development

Compound indicators that comprehensively summarize multiple facets of road safety information are increasingly recognized as valuable for policy analysis (38). This study proposes a composite set of SPIs to capture a clearer depiction of general road safety conditions compared to traditional indices, focusing primarily only on fatality rates. Previous studies have developed various SPIs to evaluate overall road safety conditions across different countries and jurisdictions (33, 34, 39, 67).

**Figure 1:**
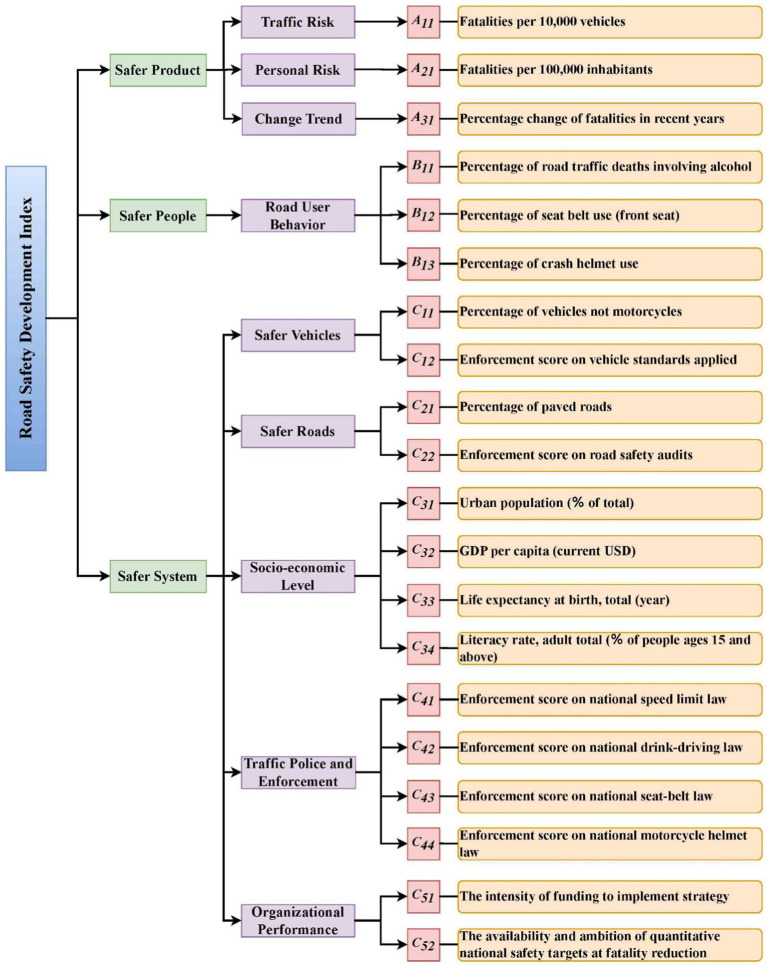
SPIs framework for this study.

The RSDI, introduced in 2005 (32), provided a solid foundation for evaluating road safety performance. It categorized influential factors into three classes: those affecting traffic exposure, accident risk, and accident severity. These SPIs were subsequently used and augmented in later research (36, 68). Our study adopted the core set of SPIs from previous research and classified them into three categories: product, people, and system. The “product” category includes direct road safety measures, such as fatalities per 100,000 inhabitants. The “people” category examines behaviors such as seat belt and helmet use. The “system” category encompasses factors impacting road safety conditions across the entire system, including vehicles, roads, socioeconomic conditions, traffic police enforcement, and organizational performance. The layered RSDI framework is presented in Figure 1.

### 3.2 Data collection

Data on SPIs for four years (2009, 2013, 2015, and 2018) were extracted from recent publications and several international databases for 11 Southeast Asian countries. Specifically, A11, A21, A31, C11, B11, B12, B13, C41, C42, C43, and C44 were sourced from the WHO (2, 69–71). Data for C31, C32, C33, and C34 were obtained from the World Bank database (72). Data for C21 were collected from the ASEAN Secretariat (73). Data for C12, C22, C51, and C52 were gathered from each country’s institutional framework published by the WHO (2, 69–71), with final scores assigned on a scale from 0 to 10 based on expert assessments. Human Development Index (HDI) values were acquired from reports by the United Nations Development Programme (74–77).

## 4 Methodology

### 4.1 Approaches

#### 4.1.1 EWM

The EWM is an objective technique for index weights, initially proposed in 1948 by Shannon (78). This method assigns greater weights to indicators with more variable data. Indicators with data distributions significantly different from a uniform distribution have lower entropy, which is interpreted as higher informativeness. Consequently, indicators with lower entropy receive higher weights.

In this study, EWM is implemented to determine the weights for the 20 SPIs.

#### 4.1.2 PROMETHEE II

PROMETHEE is a multi-criteria decision analysis method introduced by Brans (79). It provides both partial (PROMETHEE I) and complete (PROMETHEE II) rankings based on multiple indicators. PROMETHEE II is used in this study to generate a comprehensive ranking of the 11 countries from best to worst based on 20 indicators. By employing pairwise comparisons, PROMETHEE II offers a more detailed ranking compared to other multi-criteria decision analysis methods, enabling each Southeast Asian country to evaluate how its road safety conditions compare to their counterparts.

In this study, PROMETHEE II is employed to consolidate information weighted by EWM into an overarching ranking of general road safety conditions.

#### 4.1.3 DBSCAN enhanced with grid search

DBSCAN is a clustering approach based on the density of data point distribution. Proposed by Ester in 1996, it is designed to cluster data points into groups, or ϵ-neighborhoods, based on a specified radius (epsilon). Data points within this radius that meet or exceed a minimum number of neighbors (*minPts*) form a cluster; those that do not are considered noise. DBSCAN effectively identifies clusters of arbitrary shapes, including non-linear configurations (80–82). Unlike the k-means clustering algorithm, which requires specifying the number of clusters in advance, DBSCAN can detect clusters of varying shapes and sizes, including those that may be entirely enclosed by other clusters. Additionally, DBSCAN is robust to outliers due to its ability to recognize noise.

An advanced variant, hierarchical DBSCAN, has been recently introduced (83). This version builds on the traditional DBSCAN algorithm but allows for clustering of varying densities. Hierarchical DBSCAN maintains stability across different runs and parameter settings, offering consistent results even with varying density levels (84). However, it is more complex and slower than standard DBSCAN. Hierarchical clustering, an unsupervised method, can use any valid measure of distance and can determine the optimal number of clusters from the data itself (85, 86). Nonetheless, due to its high time and space complexity, it may not be suitable for large datasets.

In this study, we enhance DBSCAN with a machine learning algorithm, specifically grid search. Grid search is a hyperparameter optimization technique that systematically evaluates model performance across a grid of hyperparameter values to select the combination that yields the best results. By using grid search, we systematically test different hyperparameter combinations, training and evaluating DBSCAN with each combination on the training and validation sets. The hyperparameter combination that achieves the best performance metric on the validation set is then selected. Given the relatively small sample size (11 countries), we made modifications to achieve effective clustering. In the first round, we clustered the data points by linking each point to its closest neighbor. In the second round, we linked the groups formed in the first round by connecting those with the smallest average distance between their members. The process ceases when three distinct neighborhoods are separated from each other.

### 4.2 Model specification

In the EWM–PROMETHEE II–DBSCAN model, the weights calculated by EWM are first assigned to each indicator. Based on these weighted criteria, PROMETHEE II is then implemented to produce country scores and rankings for four separate years (2009, 2013, 2015, and 2018). Finally, DBSCAN clusters the 11 countries into three different groups for each study year according to the similarities among the indicators.

The detailed procedure of the EWM–PROMETHEE II–DBSCAN model is as follows:

**Step 1**: form a decision matrix

We assume that an MCDM problem has m alternatives (11 countries in Southeast Asia), each evaluated with n criteria (SPIs in this study). This can be succinctly represented in a matrix $R=\left(r_{ij}\right)m\times n$ , as Equation 1:


(1) $$R=\left[\begin{array}{cccc} r_{11} & r_{12} & \cdots & r_{1n}\\ r_{21} & r_{22} & \cdots & r_{2n}\\ \vdots & \vdots & \ddots & \vdots \\ r_{m1} & r_{m2} & \cdots & r_{mn} \end{array}\right]$$

**Step 2**: normalize the decision matrix

To eliminate inconsistencies in indicator sizes and directions, we normalize it using the min-max method.

For negative indicators (A11, A21, A31, and B11 in this study) using Equation 2:


(2) $$x_{ij}=\frac{r_{j\max}-r_{ij}}{r_{j\max}-r_{j\min}}$$

For positive indicators (all criteria apart from A11, A21, A31, and B11) using Equation 3:


(3) $$x_{ij}=\frac{r_{ij}-r_{j\min}}{r_{j\max}-r_{j\min}}$$

After normalization, the matrix is transformed as Equation 4:


(4) $$X=\left[\begin{array}{cccc} x_{11} & x_{12} & \cdots & x_{1n}\\ x_{21} & x_{22} & \cdots & x_{2n}\\ \vdots & \vdots & \ddots & \vdots \\ x_{m1} & x_{m2} & \cdots & x_{mn} \end{array}\right]$$

**Step 3**: assign weights to the indicators through EWM using Equations 5–7:


(5) $$p_{ij}=\frac{x_{ij}}{\overset{m}{\underset{i=1}{\sum }}x_{ij}}$$

The entropy values of the criteria are as follows:


(6) $$e_{j}=-\frac{1}{\ln\left(m\right)}\overset{m}{\underset{i=1}{\sum }}\left(p_{ij}\times \ln p_{ij}\right)$$

The weights of the indicators are given by:


(7) $$w_{j}=\frac{1-e_{j}}{\overset{n}{\underset{j=1}{\sum }}\left(1-e_{j}\right)}$$

**Step 4**: compute the preference function using Equation 8:


(8) $$P_{j}\left(x_{ij},x_{kj}\right)=\{\begin{array}{c} 0,\;x_{ij}-x_{kj}\leq 0\\ x_{ij}-x_{kj},\;x_{ij}-x_{kj}>0 \end{array}$$

**Step 5**: compute the preference index using Equation 9:


(9) $$\begin{array}{l} \prod \left(a_{i},a_{k}\right)=\overset{n}{\underset{j=1}{\sum }}w_{j}P_{j}\left(x_{ij},x_{kj}\right)\\ i,k=1,2,\ldots ,m;\;j=1,2,\ldots ,n \end{array}$$

where $a_{i}$ and $a_{k}$ denote the ith and kth alternatives, respectively, and $w_{j}$ is the weight of the jth criterion calculated in **Step 3**.

**Step 6**: compute the outranking flow

The leaving flow is given by using Equation 10:


(10) $$\Phi^{+}\left(a_{i}\right)=\overset{m}{\underset{k=1}{\sum }}\prod \left(a_{i},a_{k}\right);\;i,k=1,2,\ldots ,m$$

The entering flow is given by using Equation 11:


(11) $$\Phi^{-}\left(a_{i}\right)=\overset{m}{\underset{k=1}{\sum }}\prod \left(a_{k},a_{i}\right);\;i,k=1,2,\ldots ,m$$

**Step 7**: compute the PROMETHEE scores using Equation 12:


(12) $$\Phi \left(a_{i}\right)=\Phi^{+}\left(a_{i}\right)-\Phi^{-}\left(a_{i}\right);\;i=1,2,\ldots m$$

**Step 8**: rank the objects based on the PROMETHEE scores

A higher PROMETHEE score indicates better road safety performance.

**Step 9:** grid search for identifying the optimal parameters for DBSCAN.

A grid search in machine learning was used to identify the optimal parameters for the DBSCAN algorithm. The *ε* parameter was set to vary within a range from 0.000001 to 0.01, with an increment of 0.000001 at each step. The *minPts* parameter ranged from 2 to 10. For each possible combination of parameters, DBSCAN was run on the dataset, and its performance was evaluated using the Silhouette Coefficient (87). This evaluation involved calculating the mean distance between a sample and all other points in the same class (a), the mean distance between a sample and all other points in the nearest cluster that the sample is not a part of (b), and then computing the silhouette coefficient for each sample using Equation 13:


(13) $$s=\frac{b-a}{\max\left(a,b\right)}$$

The value of the silhouette coefficient ranges from-1 to 1, and it can be used to determine the optimal parameters (*ε* and *minPts*) by selecting the combination that maximizes the average silhouette coefficient across all data points, indicating the best overall clustering performance.

Every possible combination of these parameters was explored, and the best combination was selected based on the silhouette coefficient (88).

**Step 10**: cluster the countries.

Compute the difference between countries in an *n*-dimensional space and denote the difference by *K_i_*, as Equation 14:


(14) $$K_{i}=\left[\begin{array}{cccc} x_{i1}-x_{11} & x_{i2}-x_{12} & \cdots & x_{\mathrm{in}}-x_{1n}\\ x_{i1}-x_{21} & x_{i2}-x_{22} & \cdots & x_{\mathrm{in}}-x_{2n}\\ \vdots & \vdots & \ddots & \vdots \\ x_{i1}-x_{m1} & x_{i2}-x_{m2} & \cdots & x_{\mathrm{in}}-x_{mn} \end{array}\right]$$

The distances between nations in the multidimensional space can be computed as Equation 15:


(15) $$D_{i}=|\begin{array}{l} \sqrt{{\left(x_{i1}-x_{11}\right)}^{2}+{\left(x_{i2}-x_{12}\right)}^{2}+\cdots +{\left(x_{\mathrm{in}}-x_{1n}\right)}^{2}}\\ \sqrt{{\left(x_{i1}-x_{21}\right)}^{2}+{\left(x_{i2}-x_{22}\right)}^{2}+\cdots +{\left(x_{\mathrm{in}}-x_{2n}\right)}^{2}}\\ \;\vdots \\ \sqrt{{\left(x_{i1}-x_{m1}\right)}^{2}+{\left(x_{i2}-x_{m2}\right)}^{2}+\cdots +{\left(x_{\mathrm{in}}-x_{mn}\right)}^{2}} \end{array}|$$

Then, the countries were classified into three clusters based on these distances. We first clustered each country with its closest neighbor. If there were more than three clusters, the two clusters with the closest average distance were merged. For instance, suppose there are two groups, α containing $\left(a,b,c\right)$ and β containing $\left(x,y,z\right)$ . Thus, the distance between the two groups, $D\left(\alpha \beta \right)$ , is as Equation 16:


(16) $$D\left(\alpha \beta \right)=\frac{\begin{array}{l} D\left(\mathrm{ax}\right)+D\left(ay\right)+D\left(az\right)+D\left(bx\right)+D\left(by\right)+D\left(bz\right)\\ +D\left(cx\right)+D\left(cy\right)+D\left(cz\right) \end{array}}{9}$$

**Step 11**: determine the benchmark countries

The countries within the same group share similarities in indices and can learn from one another. The benchmark is the nation with the best safety performance score (PROMETHEE II value), achieving the highest status in road safety conditions and being labeled as “best-in-class.” Accordingly, road safety condition improvements in other countries in the group can be achieved by learning from the benchmark country.

## 5 Results and discussion

### 5.1 Empirical results

#### 5.1.1 Cross-country comparison of road safety development

Using the EWM–PROMETHEE II–DBSCAN, we computed the PROMETHEE scores for road safety advancements in 11 Southeast Asian countries over the four study years (2009, 2013, 2015, and 2018). These scores were derived by integrating the SPIs into a comprehensive index, and the results are shown in Table 1 alongside the HDI scores for each year.

**Table 1:** Country rankings for 2009–2018.

Country	ISO	2009		2013		2015		2018	
		Score	Rank	Score	Rank	Score	Rank	Score	Rank
Brunei	BN	0.566	2	5.648	1	4.498	1	1.678	1
Indonesia	ID	0.204	4	−0.373	5	−0.142	4	−0.136	5
Cambodia	KH	−0.719	11	−1.736	10	−0.970	9	−0.491	7
Laos	LA	−0.680	10	−1.354	7	−0.600	5	−0.605	11
Myanmar	MM	−0.109	6	−1.607	8	−1.283	10	−0.500	8
Malaysia	MY	−0.057	5	0.407	4	0.289	3	−0.067	4
Philippines	PH	1.460	1	−1.701	9	−0.886	8	−0.533	10
Singapore	SG	0.484	3	3.146	2	1.887	2	1.149	2
Thailand	TH	−0.461	8	−0.518	6	−0.859	7	−0.515	9
Timor-Leste	TL	−0.531	9	−2.462	11	−1.283	11	−0.221	6
Vietnam	VN	−0.157	7	0.550	3	−0.650	6	0.241	3

As shown in Table 1, some countries exhibited improving trends, while others showed deterioration. For instance, Cambodia (KH) made progress in improving road safety conditions, which is reflected in its ranking improvement from 11th to 10th, 9th, and finally 7th. Conversely, the Philippines (PH) dropped from 1st in 2009 to 10th in 2018. Brunei (BN) and Singapore (SG) consistently ranked high over the four years. However, the countries that performed poorly demonstrated significantly lower scores than the top performers, requiring more effort to enhance their road safety conditions.

#### 5.1.2 Identification of benchmark countries

The central idea of benchmarking is to learn from the best within a group of similar features. In addition to data quality, the appropriate cluster size is crucial to successful benchmarking. Considering the small sample size in this study, it was optimal to cluster the 11 Southeast Asian countries into three groups. Each group had its benchmark identified, as shown in Table 2.

**Table 2:** Grouping of countries with best-in-class across the four years.

Group	2009		2013		2015		2018	
	Country	Benchmark	Country	Benchmark	Country	Benchmark	Country	Benchmark
I	BN MY SG	BN	BN MY SG ID	BN	BN SG MY	BN	BN SG MY	BN
II	ID PH TH VN	PH	PH TH VN	VN	ID KH PH TH VN	ID	ID KH PH TH VN	VN
III	KH LA MM TL	TL	KH LA MM TL	LA	LA MM TL	LA	LA MM TL	TL

BN-Brunei, ID-Indonesia, KH-Cambodia, LA-Laos, MM-Myanmar, MY-Malaysia, PH-Philippines, SG-Singapore, TH-Thailand, TL-Timor-Leste, VN-Vietnam. Green–High level of safety performance, Yellow–Medium level of safety performance, Red–Low level of safety performance.

As shown in Table 2, Brunei (BN) consistently serves as the benchmark for the countries in Group I. Vietnam (VN) acts as the benchmark for two years (2013 and 2018) within Group II. In Group III, Timor-Leste (TL) and Laos (LA) alternately share the benchmark position over the ten years.

### 5.2 Robustness analyses

#### 5.2.1 Robustness check of ranking

In subsequent model trials, the approaches used in different segments of the model were adjusted. For ranking robustness, the tests included four phases: (1) different normalization methods (i.e., min-max, vector, and z-score) were examined, (2) different weighting methods (i.e., entropy, criteria importance through inter-criteria correlation (CRITIC), and standard deviation) were tested, (3) different aggregating methods (i.e., PROMETHEE II, TOPSIS, and RSR) were compared, and (4) different measuring methods [i.e., safety score, HDI, and Logistics Performance Index (LPI)] were contrasted.

##### 5.2.1.1 Initial stability

To test the initial stability of the proposed model, we compared the ranking results based on different normalization methods (i.e., min-max, vector, and z-score), which are classical normalization techniques in data science (89, 90).

**Figure 2:**
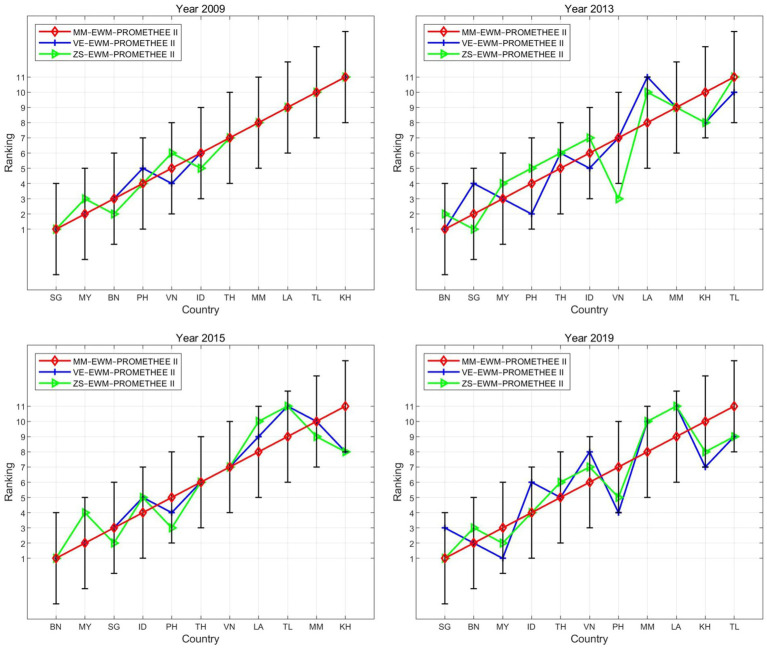
Ranking lines across different normalization methods.

As shown in Figure 2, the rankings of the countries obtained from the proposed model are consistent with those from the other two models, exhibiting similar distribution in the ranking lines. For instance, in 2009, many countries (i.e., KH, LA, MM, PH, SG, TH, and TL) maintained the same rankings across the three models. For the other three years (2013, 2015, and 2019), despite slight deviations (less than 3 ranks), the country rankings exhibited a high degree of consistency, as evidenced by the high correlation coefficients, which were generally above 0.9 (Table 3).

**Table 3:** Spearman’s correlation between rankings using various normalization methods.

Year	Models	MinMax	Vector	Z-score
2009	MinMax	1.000	0.991^**^	0.982^**^
	Vector		1.000	0.991^**^
	Z-Score			1.000
2013	MinMax	1.000	0.891^**^	0.864^**^
	Vector		1.000	0.809^**^
	Z-Score			1.000
2015	MinMax	1.000	0.927^**^	0.873^**^
	Vector		1.000	0.964^**^
	Z-Score			1.000
2018	MinMax	1.000	0.791^**^	0.891^**^
	Vector		1.000	0.936^**^
	Z-Score			1.000

* Correlation is significant at the 0.05 level (2-tailed). ** Correlation is significant at the 0.01 level (2-tailed).

##### 5.2.1.2 Internal consistency

To examine the internal consistency of the proposed model, we compared the ranking results based on different weighting methods (i.e., entropy, CRITIC, and standard deviation), which are well-established objective techniques for assigning weights to criteria (91).

**Figure 3:**
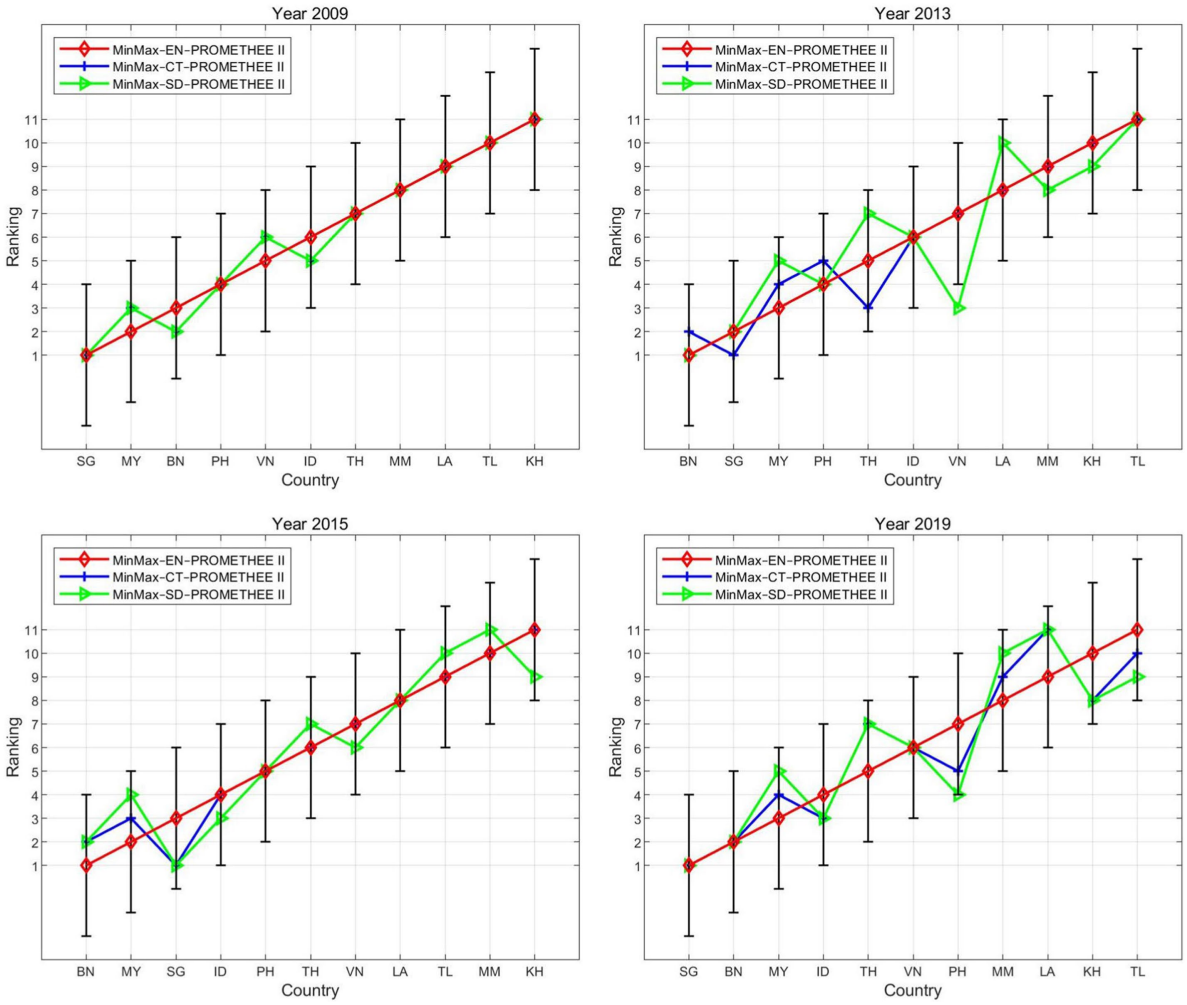
Ranking lines across different weighting methods.

As shown in Figure 3, the rankings of the countries derived from the three models exhibited a high level of consistency, with similar distributions along the ranking lines. Many countries (e.g., BN, ID, KH, LA, MM, SG, TH, and TL) maintained the same rankings across the three models for at least one year. Overall, the deviations in rankings across different models are generally less than 3, and the correlation coefficients between the rankings are typically higher than 0.9 on average (Table 4).

**Table 4:** Spearman’s correlation between rankings using various weighting methods.

Year	Models	Entropy	CRITIC	Standard deviation
2009	Entropy	1.000	0.982^**^	0.982^**^
	CRITIC		1.000	1.000^**^
	Standard deviation			1.000
2013	Entropy	1.000	0.964^**^	0.864^**^
	CRITIC		1.000	0.809^**^
	Standard deviation			1.000
2015	Entropy	1.000	0.973^**^	0.918^**^
	CRITIC		1.000	0.955^**^
	Standard deviation			1.000
2018	Entropy	1.000	0.909^**^	0.845^**^
	CRITIC		1.000	0.982^**^
	Standard deviation			1.000

* Correlation is significant at the 0.05 level (2-tailed). ** Correlation is significant at the 0.01 level (2-tailed).

##### 5.2.1.3 Horizontal reliability

To examine the horizontal reliability of the proposed model and minimize the chance of yielding inaccurate and erroneous results, we compared the results produced by other well-known classical methods. Therefore, the RSR and TOPSIS methods, two classical MCDM tools that have been useful in preceding studies concerning road safety conditions (92, 93), served as references.

**Figure 4:**
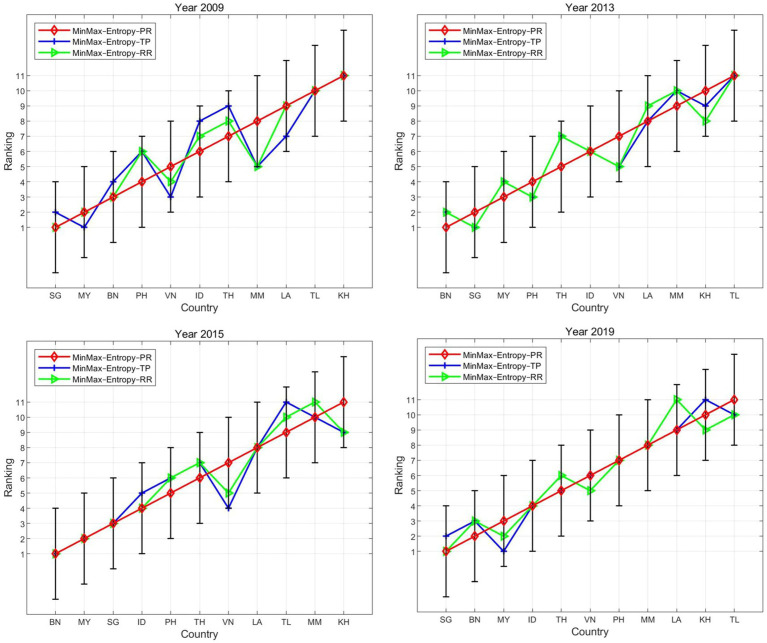
Ranking lines across different aggregation methods.

As shown in Figure 4, the rankings obtained from the proposed model and the other two models were similar for the four years. Although a slight deviation was observed, the general trend was consistent, with their points sharing a similar distribution. The correlation coefficients between the three approaches were all above 0.9 at the 0.01 significance level (Table 5). The consistency of the rankings among the three models indicates the credibility of the empirical results and the robustness of the proposed model, demonstrating its ability to gauge road safety conditions effectively.

**Table 5:** Spearman’s correlation between rankings using various aggregation methods.

Year	Models	PROMETHEE II	TOPSIS	RSR
2009	PROMETHEE II	1.000	0.905^**^	0.927^**^
	TOPSIS		1.000	0.955^**^
	RSR			1.000
2013	PROMETHEE II	1.000	936^**^	0.918^**^
	TOPSIS		1.000	0.991^**^
	RSR			1.000
2015	PROMETHEE II	1.000	0.909^**^	0.945^**^
	TOPSIS		1.000	0.982^**^
	RSR			1.000
2018	PROMETHEE II	1.000	0.964^**^	0.955^**^
	TOPSIS		1.000	0.945^**^
	RSR			1.000

* Correlation is significant at the 0.05 level (2-tailed). ** Correlation is significant at the 0.01 level (2-tailed).

##### 5.2.1.4 External dependability

To examine the external dependability of the proposed model, we compared the ranking results from the proposed model with those obtained from different measurement approaches (i.e., HDI and LPI). These are well-known composite measurement and benchmarking tools that reflect transport-related development (94–96).

**Figure 5:**
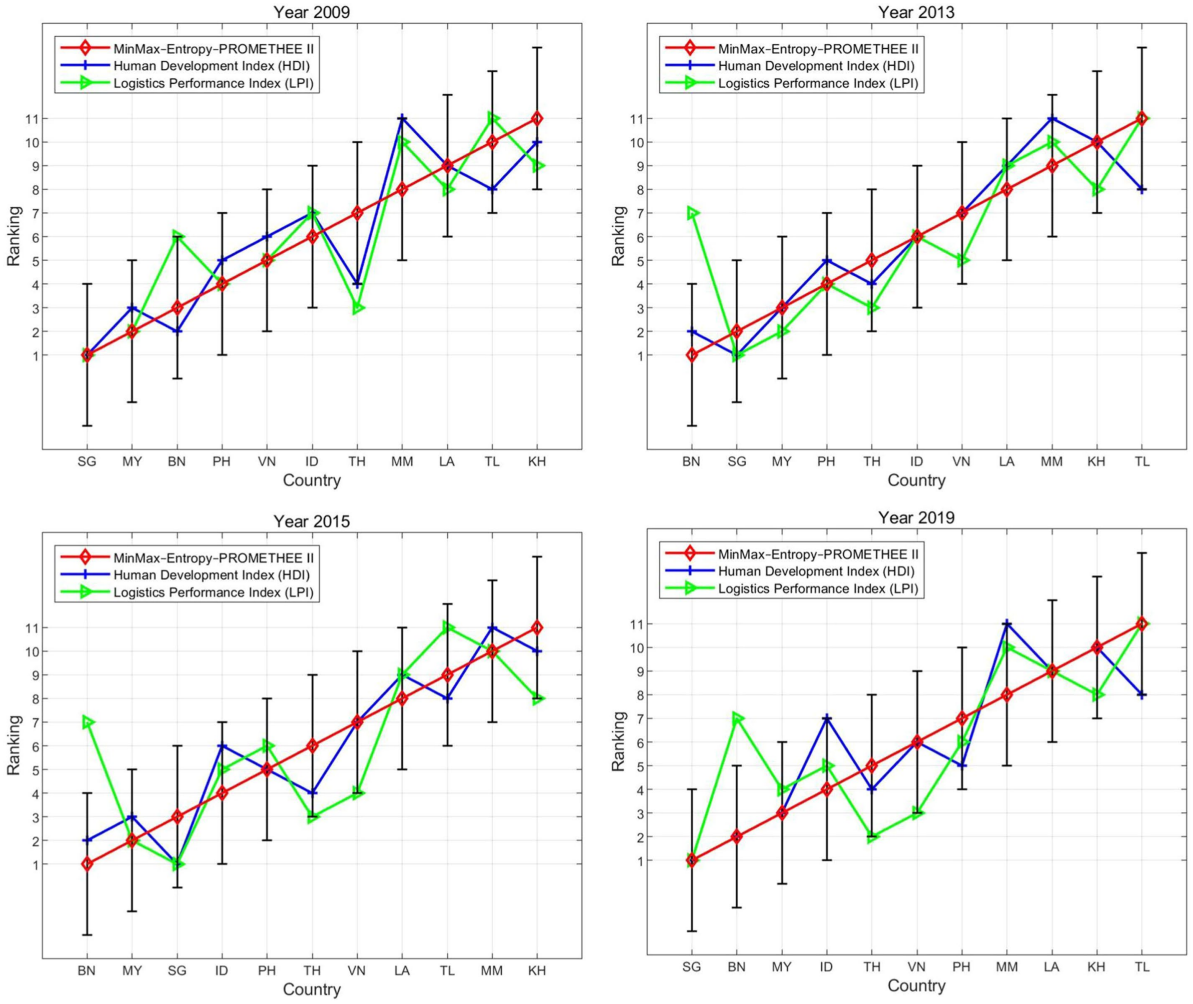
Ranking lines across different measurement methods.

As shown in Figure 5, the road safety scores (from the proposed model) were clearly positively correlated with the HDI and LPI scores. Countries with lower safety scores generally had lower HDI and LPI ranks, and countries with higher safety scores typically had higher HDI and LPI ranks. For instance, Brunei (BN) and Singapore (SG), which consistently ranked high in road safety, also had the highest HDI rankings. However, it is important to note that this relationship underscores correlation rather than causation between the HDI and the road safety index (Table 6). This suggests that road safety might be a sub-dimension of the HDI and LPI.

**Table 6:** Spearman’s correlation between rankings using various measurement methods.

Year	Models	MinMax–Entropy–PROMETHEE II	HDI	LPI
2009	MinMax–Entropy–PROMETHEE II	1.000	0.873^**^	0.836^**^
	HDI		1.000	0.855^**^
	LPI			1.000
2013	MinMax–Entropy–PROMETHEE II	1.000	0.918^**^	0.764^**^
	HDI		1.000	0.791^**^
	LPI			1.000
2015	MinMax–Entropy–PROMETHEE II	1.000	0.918^**^	0.664^*^
	HDI		1.000	0.764^**^
	LPI			1.000
2018	MM–EN–PR	1.000	0.855^**^	0.755^**^
	HDI		1.000	0.736^**^
	LPI			1.000

* Correlation is significant at the 0.05 level (2-tailed). ** Correlation is significant at the 0.01 level (2-tailed).

#### 5.2.2 Robustness check of grouping

For assessing the robustness of the grouping, two phases were conducted: (1) evaluating different grouping methods [i.e., DBSCAN, PCA, and Fuzzy C-Means (FCM)] and (2) comparing different measurement methods (i.e., safety score, HDI, and LPI).

##### 5.2.2.1 Horizontal reliability

To verify the reliability of the clusters, PCA and FCM, which are commonly used in transportation grouping, were utilized as alternatives, as shown in Table 7.

**Table 7:** Clustering of countries based on the proposed model, PCA, and FCM.

Country	2009			2013			2015			2018		
	DBSCAN	PCA	FCM	DBSCAN	PCA	FCM	DBSCAN	PCA	FCM	DBSCAN	PCA	FCM
BN	I	I	I	I	I	I	I	I	I	I	I	I
MY	I	I	I	I	I	I	I	I	I	I	I	I
SG	I	I	I	I	I	I	I	I	I	I	I	I
ID	II	II	II	I	II	II	II	II	II	II	I	II
PH	II	II	II	II	II	II	II	II	II	II	II	II
TH	II	II	II	II	II	II	II	II	II	II	II	II
VN	II	II	II	II	II	II	II	II	II	II	II	II
KH	III	III	III	III	III	III	II	III	III	II	III	III
LA	III	II	III	III	III	III	III	III	III	III	III	III
MM	III	II	II	III	III	II	III	III	II	III	III	II
TL	III	III	III	III	III	III	III	III	III	III	III	III

Green–High level of safety performance, Yellow–Medium level of safety performance, Red–Low level of safety performance.

As shown in Table 7, the clustering results based on the proposed method align well with those from PCA and FCM across the four years. In 2013, the groupings from all methods were identical. The similarity in grouping confirmed the robustness and credibility of the proposed model. In 2009, 2013, and 2015, the countries identified as the poorest performers by both approaches were consistent (i.e., Timor-Leste (TL) and Cambodia (KH) in 2009; Laos (LA), Myanmar (MM), Cambodia (KH), and Timor-Leste (TL) in 2013; and Laos (LA), Myanmar (MM), Cambodia (KH), and Timor-Leste (TL) in 2015). These results indicate that Cambodia (KH) and Timor-Leste (TL) consistently performed poorly in safety among ASEAN countries, with Timor-Leste (TL) remaining in the worst group across all years. However, Cambodia (KH) showed improvement over the years, achieving a middle-ranking position in 2018 based on DBSCAN clustering.

In 2009, the best-performing group identified by the DBSCAN clustering method was notably larger compared to other years. This is attributed to the similar road safety conditions across countries in 2009. Since DBSCAN clusters are based on the distance between data points in a multidimensional space, it is likely to group a large number of similar countries together in 2009.

Meanwhile, some inconsistencies were identified between country rankings and their assigned groups. This can be explained by the clustering algorithm and methodology employed. Clustering involves grouping countries based on similarities, with no weights applied to the indices, and normalized data to eliminate scale differences. Conversely, rankings are calculated using weighted data from Step 4. Such differences in the ranking and clustering outcomes may result from variations in the calculation processes.

Overall, the comparison of results confirms the robustness of the proposed methodology and its practical applicability. The model yields more comprehensive insights compared to PCA, which reduces data dimensions and allows for a focus on just the two most influential indices. This makes the model more useful and adaptable for policymakers and decision-makers.

##### 5.2.2.2 External dependability

To assess the external dependability of the proposed model, we compared the clustering results from the proposed model with those based on HDI and LPI, as shown in Table 8.

**Table 8:** Clustering of countries based on the proposed model, HDI, and LPI.

Country	2009			2013			2015			2018		
	Safety score	HDI	LPI	Safety score	HDI	LPI	Safety score	HDI	LPI	Safety score	HDI	LPI
BN	I	I	I	I	I	I	I	I	I	I	I	I
ID	II	II	II	II	II	II	II	II	II	II	II	II
MY	I	I	I	I	I	I	I	I	I	I	I	I
PH	II	II	I	II	II	II	II	II	II	I	I	I
SG	I	I	I	I	I	I	I	I	I	I	I	I
TH	II	II	II	II	II	II	II	II	II	II	II	II
VN	II	II	II	II	II	II	II	II	II	II	II	II
LA	III	III	III	III	III	III	III	III	III	III	III	III
MM	II	II	II	III	III	II	III	III	II	II	III	II
KH	III	III	III	III	III	III	III	III	III	II	III	III
TL	III	III	III	III	III	III	III	III	III	III	III	III

Green–High level of safety performance, Yellow–Medium level of safety performance, Red–Low level of safety performance.

As indicated in Table 8, the clustering results based on the safety scores from the proposed method align closely with those obtained from PCA and FCM across the four years. This consistency further validates the dependability of the proposed model and underscores the role of safety-related factors as a sub-dimension of the HDI and LPI.

## 6 Policy implications

### 6.1 Longitudinal overall progress

**Figure 6:**
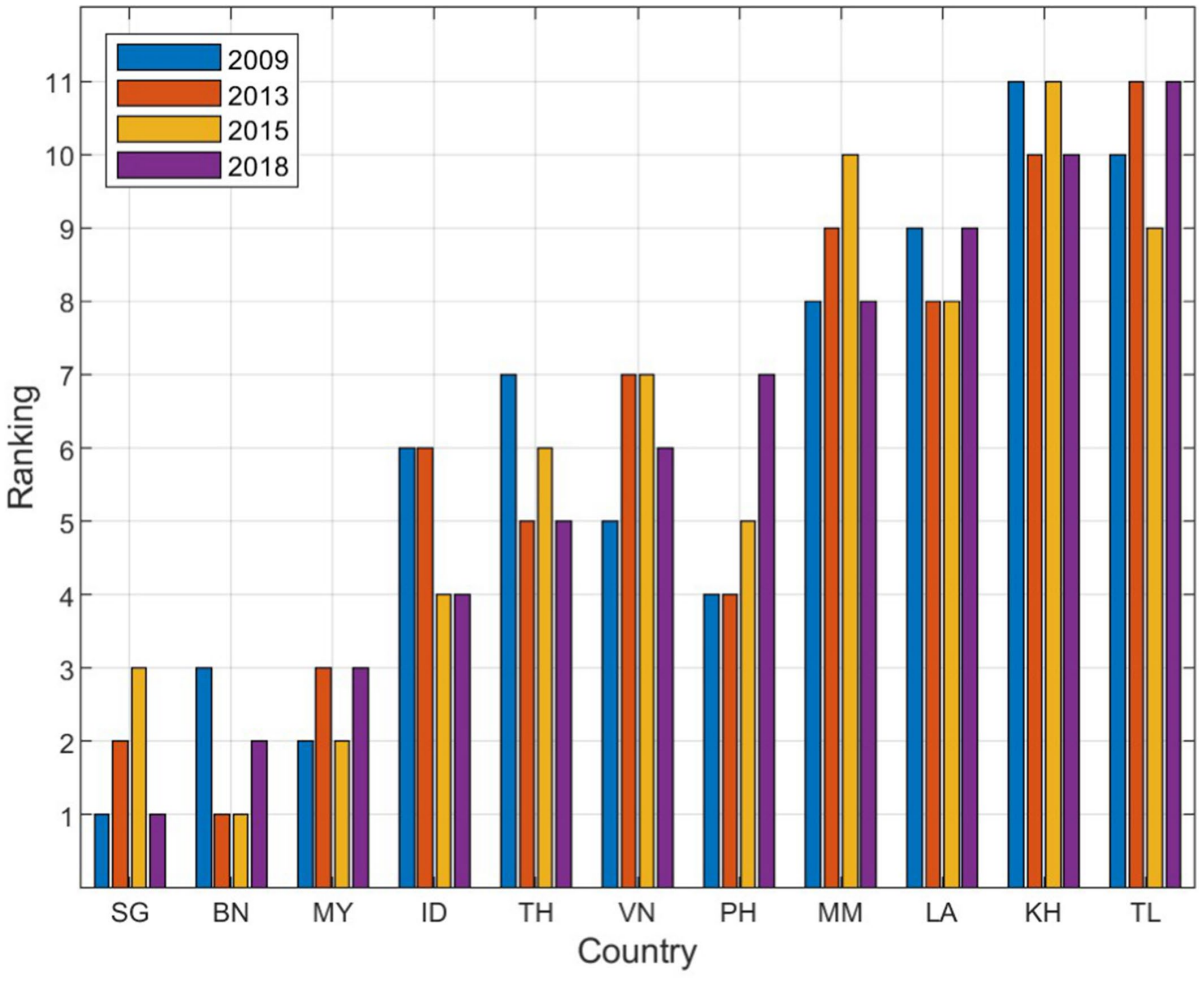
Country rankings from 2009 to 2018.

Figure 6 illustrates the country rankings on the PROMETHEE index from 2009 to 2018.

As shown in Figure 6, Singapore (SG), Brunei (BN), and Malaysia (MY) consistently ranked among the top performers across the four years. The sustained high rankings of Singapore (SG) and Brunei (BN) can be attributed to their advanced national development across multiple sectors and their ongoing efforts to uphold excellent road safety conditions.

Conversely, Timor-Leste (TL), Cambodia (KH), and Laos (LA) consistently ranked at the lower end. Specifically, Cambodia (KH) ranked lowest in 2009 and 2015, while Timor-Leste (TL) was at the bottom in 2013 and 2018. Laos (LA) showed some improvement from 9th to 8th place between 2009 and 2015 but fell back to 9th in 2018. This decline is likely related to serious flooding caused by a dam collapse in July 2018 (97).

The countries with moderate performance showed varied trends. For instance, Indonesia (ID) ranked 6th in 2009 but has generally improved over time. Thailand (TH) advanced from 7th place in 2009 to 5th place in 2018, demonstrating a clear effort to enhance road safety. Conversely, Myanmar (MM) experienced a decline, dropping from 8th place in 2009 to 10th in 2013. The Philippines (PH) experienced a notable regression from 2013 to 2018, falling from 4th to 7th place. This decline is supported by the original data, which reveals a significant increase in all three criteria from 2009 to 2013: A11 (fatalities per 10,000 vehicles) increased from 2.1 to 10.5; A21 (fatalities per 100,000 inhabitants) increased from 1.3 to 7.4; and A31 (percentage change of fatalities in recent years) jumped from 165.5 to 585.7. This sudden rise in these indices likely explains for the drop in the Philippine’s (PH’s) ranking. Vietnam (VN) also showed a downward trend, moving from 5th to 7th place between 2009 to 2015. Overall, road safety conditions across Southeast Asia have experienced significant changes from 2009 to 2018.

Compared to previous studies lacking vertical comparison (29, 98), this longitudinal analysis provides valuable insights into long-term changes and the effectiveness of interventions. It offers a comprehensive perspective on progress over time, aiding in decision-making and continuous improvement.

### 6.2 Decomposition of achievement differences

**Figure 7:**
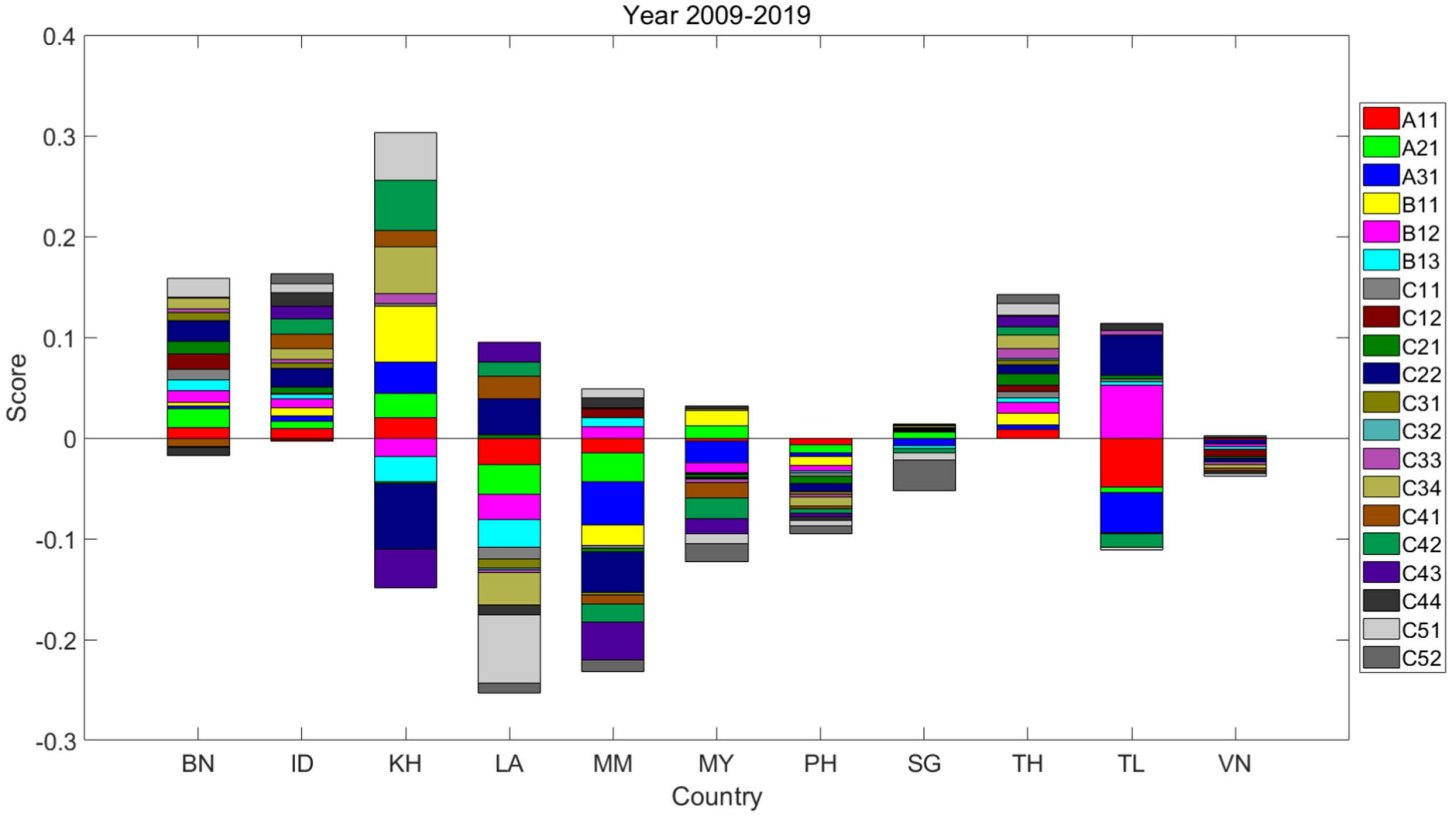
Decomposition of achievement differences between 2009 and 2019.

Decomposing the achievement difference involves breaking down the variations between different achievements into various factors or components (Figure 7). This approach helps identify the specific factors contributing to the observed discrepancies.

As shown in Figure 7, countries such as Brunei (BN), Indonesia (ID), Cambodia (KH), and Thailand (TH) have shown the most significant improvements in transport safety over the decade from 2009 to 2019. In contrast, Timor-Leste (TL) has seen minimal to no change in this area. Laos (LA), Myanmar (MM), Malaysia (MY), and the Philippines (PH) have experienced notable declines in their transport safety performance, while Singapore (SG) and Vietnam (VN) have seen slight decreases during the same period.

Key indicators A11, A21, and A31, which measure fatalities, are crucial for enhancing transportation safety. These metrics are also indispensable for policymakers assessing the effectiveness of past transport policies. Over the specified period, four countries showed an increase in A11, five countries saw an improvement in A21, and four countries experienced a rise in A31. Countries with notable improvements, namely Brunei (BN), Indonesia (ID), Cambodia (KH), and Thailand (TH), have all made progress in A11. Brunei (BN) and Cambodia (KH) have achieved significant advancements in A21, with Cambodia (KH) also demonstrating substantial progress in A31. In contrast, Thailand (TL) has experienced significant declines in both A11 and A31 and Myanmar (MM) has recorded substantial declines across all three indicators.

Indicators B11, B12, and B13, which measure road user behavior, are significant influences on transport safety. B11 measures alcohol consumption, a primary contributor to traffic-related fatalities. Five countries reported improvements in B11, while two witnessed declines. Cambodia (KH) has made considerable improvements, while Myanmar (MM) has seen the most significant deterioration. The use of seat belts and motorcycle helmets, measured by B12 and B13, respectively, is crucial for individual safety. Five countries have shown an upward trend in compliance with both B12 and B13, whereas Cambodia (KH) and Laos (LA) have experienced notable declines. Thailand (TL) has made significant progress in B12. Singapore (SG) and Vietnam (VN) did not exhibit significant changes in these behavioral safety indicators.

Indicators C11 and C12 pertain to vehicle safety, which is instrumental in reducing traffic accidents and safeguarding individuals. Most countries have shown minimal variation in C11, which assesses the percentage of non-motorized vehicles, and C12, which evaluates the enforcement of vehicle safety standards. Brunei (BN) and Thailand (TH) have exhibited minor improvements in these two domains, while Laos (LA) has experienced a slight decrease in C11.

Indicators C21 and C22, which focus on safer road infrastructure, are pivotal for vehicle operation and overall transport safety. Six countries reported improvements in C21, which measures the percentage of paved roads, with Cambodia (KH) exhibiting the most notable progress. However, Myanmar (MM), Malaysia (MY), the Philippines (PH), and Timor-Leste (TL) have seen declines in this indicator. In terms of road safety audits, represented by C22, five countries have improved their enforcement scores. Laos (LA) and Timor-Leste (TL) made significant progress, while Cambodia (KH) and Myanmar (MM) experienced considerable regressions.

Indicators C31, C32, C33, and C34 are linked to a country’s socioeconomic status, which affects the government’s ability to invest in road safety infrastructure and shapes public awareness of transport safety. C34, representing the adult literacy rate, increased in four countries, with Cambodia (KH) showing the most significant improvement and Laos (LA) experiencing the most pronounced decline. GDP *per capita*, indicated by C32, remained relatively stable across all countries. Furthermore, a slight increase was observed in C31, representing the urban population, and C33, which measures life expectancy at birth, in four countries.

Indicators C41, C42, C43, and C44 focus on evaluating traffic policies and the enforcement of road safety laws. Three countries have registered improvements in C41, which assesses the enforcement of national speed limit laws, with Laos (LA) achieving the most significant enhancement. Conversely, five countries have experienced a slight decline in this area. Regarding C42, which measures the enforcement of national drink-driving laws, five countries have shown progress, with Cambodia (KH) leading in improvement, while five countries have demonstrated a decrease in performance. An increase in C43, which evaluates the enforcement of national seat belt laws, was observed in three countries, whereas Cambodia (KH) and Myanmar (MM) have faced considerable setbacks. The enforcement of national motorcycle helmet laws, indicated by C44, remained relatively stable, with Indonesia (ID) and Myanmar (MM) experiencing slight improvements, while Brunei (BN) and Laos (LA) showed minor declines.

Indicators C51 and C52 represent the organizational commitment and effectiveness in bolstering transport safety. C51 measures the level of funding allocated to implementing safety strategies, with five countries reporting increases; Cambodia (KH) exhibited the most substantial progress, while Laos (LA) experienced the greatest decline. Only two countries experienced slight improvements in C52, which assesses the availability and ambition of national safety targets for fatality reduction. Meanwhile, five countries have shown a downturn in this area, with Singapore (SG) demonstrating the most significant regression.

Compared to previous studies that did not systematically decompose achievement differences (13, 99), this analysis provides policymakers with a deeper understanding of the underlying factors driving performance disparities and facilitates targeted actions to address these issues effectively.

### 6.3 Benchmarking within the groups

**Figure 8:**
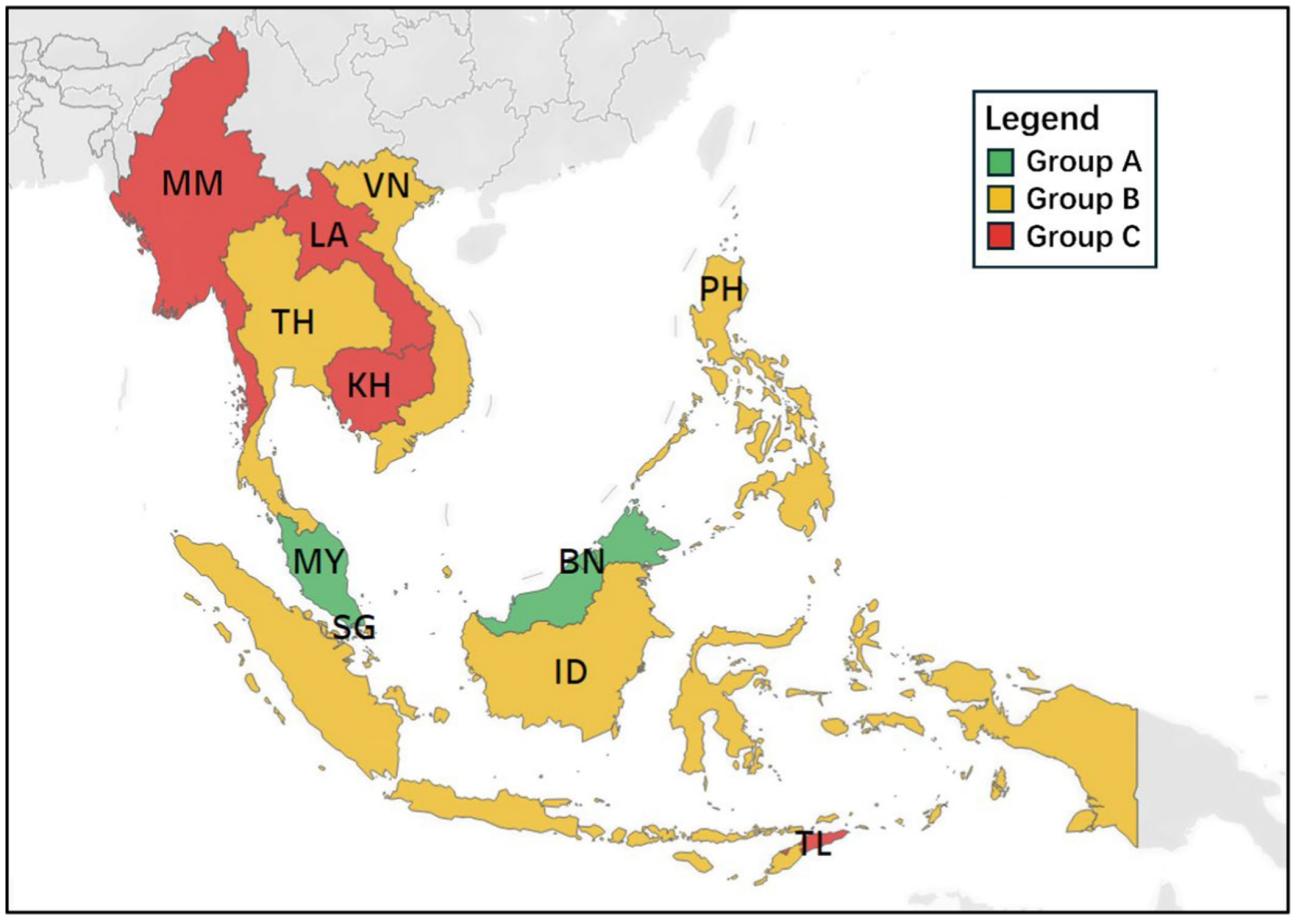
Geographical distribution of Southeast Asia countries regarding road safety progress.

**Figure 9:**
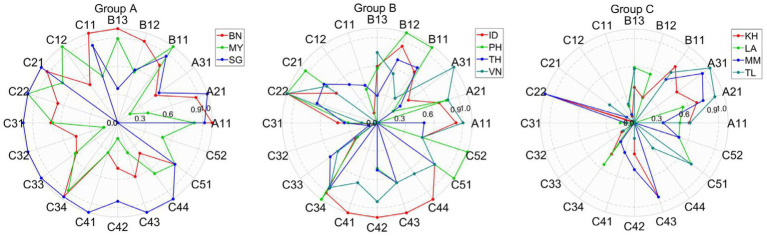
Benchmarking in SPIs within each group.

To enhance policymaking, countries facing poor road safety conditions should learn from those with better performance (see Figures 8, 9). Benchmarking is essential to this progress, as it helps identify best practices, areas for improvement, and opportunities for optimization within each group. Establishing an effective benchmarking system is vital for the improvement of road safety conditions in Southeast Asian countries.

As shown in Figures 8, 9, group A includes three leading countries, Brunei (BN), Malaysia (MY), and Singapore (SG), which achieved the highest scores based on the proposed model, highlighting their superior road safety performance in 2019. The radar chart reveals that Singapore (SG) excels in most SPIs (C21, C22, C31, C32, C33, C34, C41, C42, C43, C44, C51, A21, and A31). Brunei (BN) and Malaysia (MY) should, therefore, draw from SG’s expertise in these indicators. Malaysia (MY) outperforms in C12, B11, and C52, suggesting that other countries in Group A can benefit from Malaysia’s (MY’s) practices in these indicators. Brunei (BN) leads in C11, B13, B12, and A11, suggesting its strength in these indicators.

In group B, Indonesia (ID) is the top performer in C22, C41, C42, C43, and C44. Countries within this group should adopt successful strategies from Indonesia (ID), which has the highest score for these indicators.

For group C, which demonstrated the poorest road safety performance in 2019, the most effective approach is to adopt best practices from countries with the highest performance in each indicator. For instance, as Timor-Leste (TL) leads in C51, A21, and A31, other countries in group C should seek guidance from Timor-Leste (TL) in these indicators.

Unlike previous studies (15, 100), this approach highlights best practices, establishes performance benchmarks, and identifies areas needing improvement. It is particularly effective for assessing and improving road safety across different regions, cities, or countries with similar characteristics.

## 7 Conclusions and further research

### 7.1 Concluding remarks

This study introduces a reliable and stable MCDM methodology, specifically the EWM–PROMETHEE II–DBSCAN model, to assess overall road safety progress and support governments and legislators in formulating appropriate measures for road traffic injury prevention and control. Through a case study on road safety engineering in Southeast Asia, the empirical findings confirm the robustness of the proposed model, demonstrating its reliability, efficiency, and practical applicability in addressing real-world MCDM challenges in road safety. With reliable data, the model provides results that accurately reflect road safety progress for a given year, providing countries with a clear understanding of their road conditions. The benchmarking process enables countries to identify high-performing models from which less advanced countries can learn and improve. This approach transforms the abstract concept of improving road safety into a tangible and actionable goal by emulating successful models. Such a process not only inspires political action but also reduces the potential waste of resources and minimizes the risk of political failure in public health.

Compared to other methodologies, the proposed model is more straightforward in mathematical processes, superior in both modern nonparametric and classical parametric estimations, and adequately clear for practical application, thereby reducing barriers for officials and decision-makers in implementing effective policies. Overall, this study makes three significant contributions:

- The study constructs a set of SPIs for measuring road safety achievements in Southeast Asia, laying the foundation for regional safety action plans.
- The proposed model integrates weighting, aggregating, grouping, and benchmarking into a cohesive and refined framework. It incorporates a machine learning algorithm (grid search) into DBSCAN, enhancing the clustering efficiency by accurately calculating optimal values for neighborhood radius (ε) and 𝑚𝑖𝑛𝑃𝑡𝑠, thus addressing issues related to model failure and parameter selection in DBSCAN.
- The study provides Southeast Asian policymakers and government officials with a practical toolkit for implementing targeted road safety strategies. It offers a step-by-step framework that is accessible even to those with limited specialized knowledge in road safety.

### 7.2 Limitations and future research

Despite the robust results obtained, the proposed model has two primary limitations. First, the EWM is an objective technique that assigns greater weight to indices with more varied data, without considering their practical significance. This issue can be alleviated by allowing experts to manually adjust the weight of each index. Second, a substantial proportion of the data was directly obtained from WHO reports (2, 69–71). However, the statistics in these reports, such as the percentage of helmet use, may not be consistent in terms of source or year of representation. This discrepancy can introduce measurement bias, complicating the comparison of overall road safety progress across countries. Therefore, the results of the model should be interpreted with caution. Fortunately, re-running the model without the helmet use indicator did not exhibit any significant threats to the model outputs. To improve monitoring and evaluation, it is essential for Southeast Asian countries to collect more detailed data on road safety, such as the percentage of paved roads and helmet use.

This study highlights several avenues for future research. First, the set of SPIs should be periodically reviewed and updated. As autonomous vehicles become more prevalent and traffic environments become increasingly complex, SPIs need to evolve to reflect these changes. Second, with advancements in big data and artificial intelligence, data-driven approaches using machine learning are being broadly employed in road safety analyses. Future research should focus on investigating machine learning techniques for enhanced data-driven decision-making in road safety. In summary, improving road safety is an ongoing challenge that requires sustained effort, substantial political will, and financial resources. This endeavor should be viewed as a long-term, continuous process involving regular updates and exploration of innovative approaches.

## Data Availability

The raw data supporting the conclusions of this article will be made available by the authors, without undue reservation.

## References

[1] World Health Organization. Global status report on road safety 2023. Geneva: World Health Organization (2023).

[2] World Health Organization. Global status report on road safety 2018: summary. Geneva: World Health Organization (2018).

[3] Nationen V. The Sustainable Development Goals Report 2016. New York: UN (2020).

[4] World Health Organization. Health at a glance: Asia/Pacific 2020: measuring progress towards universal health coverage. Paris: OECD Publishing (2020).

[5] Alvarez PA Ishizaka A Martínez L. Multiple-criteria decision-making sorting methods: a survey. Expert Syst Appl. (2021) 183:115368. doi: 10.1016/j.eswa.2021.115368

[6] Lopez LM Ishizaka A Qin J Alvarez-Carrillo PA. Multi-criteria decision-making sorting methods: applications to real-world problems Elsevier (2023). 268 p.

[7] Thakkar JJ. Multi-criteria decision making. Studies in Systems, Decision and Control. Singapore: Springer. (2021). 390 p.

[8] Chakraborty S Chatterjee P Das PP. Multi-criteria decision-making methods in manufacturing environments: models and applications. New York: CRC Press (2023).

[9] Wang X Hou B Teng Y Yang Y Zhang X Sun L. Reformative ROCOSD–ORESTE–LDA model with an MLP neural network to enhance decision reliability. Knowl-Based Syst. (2024) 286:111384. doi: 10.1016/j.knosys.2024.111384

[10] Zhou Z Zhang Y Zhang Y Hou B Mei Y Wu P. Advanced CRITIC–GRA–GMM model with multiple restart simulation for assuaging decision uncertainty: an application to transport safety engineering for OECD members. Adv Eng Inform. (2024) 60:102373. doi: 10.1016/j.aei.2024.102373

[11] Egilmez G McAvoy D. Benchmarking road safety of U.S. states: a DEA-based Malmquist productivity index approach. Accid Anal Prev. (2013) 53:55–64. doi: 10.1016/j.aap.2012.12.038, PMID: 23376545

[12] Chen F Wang J Deng Y. Road safety risk evaluation by means of improved entropy TOPSIS–RSR. Saf Sci. (2015) 79:39–54. doi: 10.1016/j.ssci.2015.05.006

[13] Zhu J-H Chen J Li GF Shuai B. Using cross efficiency method integrating regret theory and WASPAS to evaluate road safety performance of Chinese provinces. Accid Anal Prev. (2021) 162:106395. doi: 10.1016/j.aap.2021.106395 34563647

[14] Nghiem S Connelly LB. Benchmarking road traffic safety across OECD countries a distance function approach. JTEP. (2015) 49:539–59.

[15] Shen Y Hermans E Bao Q Brijs T Wets G. Towards better road safety management: lessons learned from inter-national benchmarking. Accid Anal Prev. (2020) 138:105484. doi: 10.1016/j.aap.2020.105484 32105839

[16] Khan MN Das S. Advancing traffic safety through the safe system approach: a systematic review. Accid Anal Prev. (2024) 199:107518. doi: 10.1016/j.aap.2024.107518, PMID: 38422878

[17] Khattak AJ Ahmad N Wali B Dumbaugh E. A taxonomy of driving errors and violations: evidence from the naturalistic driving study. Accid Anal Prev. (2021) 151:105873. doi: 10.1016/j.aap.2020.105873 33360090

[18] Alonso F Useche SA Valle E Esteban C Gene-Morales J. Could road safety education (RSE) help parents protect children? Examining their driving crashes with children on board. Int J Environ Res Public Health. (2021) 18:3611. doi: 10.3390/ijerph18073611 33807181 PMC8037421

[19] Fisa R Musukuma M Sampa M Musonda P Young T. Effects of interventions for preventing road traffic crashes: an overview of systematic reviews. BMC Public Health. (2022) 22:513. doi: 10.1186/s12889-021-12253-y, PMID: 35296294 PMC8925136

[20] Albalate D Fageda X. On the relationship between congestion and road safety in cities. Transp Policy. (2021) 105:145–52. doi: 10.1016/j.tranpol.2021.03.011

[21] Tang T Guo Y Zhou X Labi S Zhu S. Understanding electric bike riders’ intention to violate traffic rules and accident proneness in China. Travel Behav Soc. (2021) 23:25–38. doi: 10.1016/j.tbs.2020.10.010

[22] Commandeur JJF Wesemann P Bijleveld F Chhoun V Sann S. Setting road safety targets in Cambodia: a methodological demonstration using the latent risk time series model. J Adv Transp. (2017) 2017:5798174. doi: 10.1155/2017/5798174

[23] Gutierrez H Mitra S Neki K Mbugua LW Balasubramaniyan R Winer M. Comparing estimates of road traffic deaths and non-fatal road traffic injuries in Cambodia. Inj Prev. (2022) 28:340–6. doi: 10.1136/injuryprev-2021-044504, PMID: 35149595

[24] Kitamura Y Hayashi M Yagi E. Traffic problems in Southeast Asia featuring the case of Cambodia's traffic accidents involving motorcycles. IATSS Res. (2018) 42:163–70. doi: 10.1016/j.iatssr.2018.11.001

[25] Eusofe Z Evdorides H. Assessment of road safety management at institutional level in Malaysia: a case study. IATSS Res. (2017) 41:172–81. doi: 10.1016/j.iatssr.2017.03.002

[26] Kamaluddin NA Abd Rahman MF Várhelyi A. Matching of police and hospital road crash casualty records – a data-linkage study in Malaysia. Int J Inj Control Saf Promot. (2019) 26:52–9. doi: 10.1080/17457300.2018.1476385, PMID: 29806792

[27] Haque MO Haque TH. Evaluating the effects of the road safety system approach in Brunei. Transp Res A Policy Pract. (2018) 118:594–607. doi: 10.1016/j.tra.2018.08.017

[28] Jameel AK Evdorides H. Assessment of road safety performance for southeast Asian countries. J Soc Autom Eng Malaysia. (2021) 3:246–59. doi: 10.56381/jsaem.v3i3.125

[29] Shah S Ahmad N Shen Y Pirdavani A Basheer MA Brijs T. Road safety risk assessment: an analysis of transport policy and management for low-, middle-, and high-income Asian countries. Sustain For. (2018) 10:389. doi: 10.3390/su10020389

[30] Chen F Zhu Y Zu J Lyu J Yang J. Appraising road safety attainment by CRITIC-ELECTRE-FCM: a policymaking support for Southeast Asia. Transp Policy. (2022) 122:104–18. doi: 10.1016/j.tranpol.2022.04.014

[31] Papadimitriou E Yannis G Bijleveld F Cardoso JL. Exposure data and risk indicators for safety performance assessment in Europe. Accid Anal Prev. (2013) 60:371–83. doi: 10.1016/j.aap.2013.04.040 23769621

[32] Al-Haji G. Towards a road safety development index (RSDI). Development of an international index to measure road safety performance, in Linköping studies in science and technology, licentiate thesis, no. 1174. Development of science and technology, Linköping University. (2005), Linköping University Electronic Press

[33] Bax C. Wesemann P Gitelman V Shen Y Goldenbeld C Hermans E. Developing a road safety index. Deliverable 4.9 of the EC FP7 project DaCoTA. (2012).

[34] Wegman F Commandeur J Doveh E Eksler V Gitelman V Hakkert S. SUNflowerNext: Towards a composite road safety performance index. Leidschendam: SWOV Institute for Road Safety Research (2008).

[35] Hermans E. A methodology for developing a composite road safety performance index for cross-coountry comparison. Limburg: UHasselt Diepenbeek (2009).

[36] Chen F Wang J Wu J Chen X Zegras PC. Monitoring road safety development at regional level: a case study in the ASEAN region. Accid Anal Prev. (2017) 106:437–49. doi: 10.1016/j.aap.2017.07.016, PMID: 28735179

[37] Shen Y Hermans E Bao Q Brijs T Wets G. Serious injuries: an additional Indicator to fatalities for road safety benchmarking. Traffic Inj Prev. (2015) 16:246–53. doi: 10.1080/15389588.2014.930831 24912069

[38] Tešić M Hermans E Lipovac K Pešić D. Identifying the most significant indicators of the total road safety performance index. Accid Anal Prev. (2018) 113:263–78. doi: 10.1016/j.aap.2018.02.003, PMID: 29453159

[39] Al-Haji G. Road safety development index: Theory, philosophy and practice. Linköping: Linköping University Electronic Press (2007).

[40] Hermans E Van den Bossche F Wets G. Combining road safety information in a performance index. Accid Anal Prev. (2008) 40:1337–44. doi: 10.1016/j.aap.2008.02.004 18606264

[41] Hakkert A. Gitelman V. Vis M., Road safety performance indicators: theory. Deliverable D3. 6 of the EU FP6 project SafetyNet. (2007).

[42] Shen Y Hermans E Brijs T Wets G Vanhoof K. Road safety risk evaluation and target setting using data envelopment analysis and its extensions. Accident Anal Prev. (2012) 48:430–41. doi: 10.1016/j.aap.2012.02.020, PMID: 22664709

[43] Gitelman V Levi S Doveh E Endy-Findling L. Developing a composite index of child road safety in a municipality. Open J Safety Sci Technol. (2013) 3:18–30. doi: 10.4236/ojsst.2013.32003

[44] Shbeeb L. Road safety performance index: a tool for crash prediction. Cogent Eng. (2022) 9:2124637. doi: 10.1080/23311916.2022.2124637

[45] Zhu G-N. Design concept evaluation considering information reliability, uncertainty, and subjectivity: an integrated rough-Z-number-enhanced MCGDM methodology. Adv Eng Inform. (2022) 54:101796. doi: 10.1016/j.aei.2022.101796

[46] Tumsekcali E Ayyildiz E Taskin A. Interval valued intuitionistic fuzzy AHP-WASPAS based public transportation service quality evaluation by a new extension of SERVQUAL model: P-SERVQUAL 4.0. Expert Syst Appl. (2021) 186:115757. doi: 10.1016/j.eswa.2021.115757

[47] Baydaş M Pamučar D. Pamučar determining objective characteristics of MCDM methods under uncertainty: an exploration study with financial data. Mathematics. (2022) 10:71115. doi: 10.3390/math10071115

[48] Kannan D Moazzeni S Darmian S Afrasiabi A. A hybrid approach based on MCDM methods and Monte Carlo simulation for sustainable evaluation of potential solar sites in east of Iran. J Clean Prod. (2021) 279:122368. doi: 10.1016/j.jclepro.2020.122368

[49] Markatos DN Malefaki S Spiros G. Pantelakis sensitivity analysis of a hybrid MCDM model for sustainability assessment—an example from the aviation industry. Aerospace. (2023) 10:385. doi: 10.3390/aerospace10040385

[50] Torkayesh AE Ecer F Pamucar D Karamaşa Ç. Comparative assessment of social sustainability performance: integrated data-driven weighting system and CoCoSo model. Sustain Cities Soc. (2021) 71:102975. doi: 10.1016/j.scs.2021.102975

[51] Bureš V Cabal J Čech P Mls K Ponce D. The influence of criteria selection method on consistency of pairwise comparison. Mathematics. (2020) 8:2200. doi: 10.3390/math8122200

[52] Liu Y Eckert CM Earl C. A review of fuzzy AHP methods for decision-making with subjective judgements. Expert Syst Appl. (2020) 161:113738. doi: 10.1016/j.eswa.2020.113738

[53] Liang F Brunelli M Rezaei J. Consistency issues in the best worst method: measurements and thresholds. Omega. (2020) 96:102175. doi: 10.1016/j.omega.2019.102175

[54] Hermans E Van den Bossche F Wets G. Uncertainty assessment of the road safety index. Reliabil Eng Syst Safety. (2009) 94:1220–8. doi: 10.1016/j.ress.2008.09.004

[55] Pelissari R Oliveira MC Abackerli AJ Ben-Amor S Assumpção MRP. Techniques to model uncertain input data of multi-criteria decision-making problems: a literature review. Int Trans Oper Res. (2021) 28:523–59. doi: 10.1111/itor.12598

[56] Morais C Estrada-Lugo HD Tolo S Jacques T Moura R Beer M. Robust data-driven human reliability analysis using credal networks. Reliabil Eng Syst Safety. (2022) 218:107990. doi: 10.1016/j.ress.2021.107990

[57] Chen ZS Zhang X Rodriguez RM Pedrycz W Martinez L Skibniewski MJ. Expertise-structure and risk-appetite-integrated two-tiered collective opinion generation framework for large-scale group decision making. IEEE Trans Fuzzy Syst. (2022) 30:5496–510. doi: 10.1109/TFUZZ.2022.3179594

[58] Barzkar A Najafzadeh M Homaei F. Evaluation of drought events in various climatic conditions using data-driven models and a reliability-based probabilistic model. Nat Hazards. (2022) 110:1931–52. doi: 10.1007/s11069-021-05019-7

[59] Najafzadeh M Homaei F Mohamadi S. Reliability evaluation of groundwater quality index using data-driven models. Environ Sci Pollut Res. (2022) 29:8174–90. doi: 10.1007/s11356-021-16158-6, PMID: 34482479

[60] Cui H Dong S Hu J Chen M Hou B Zhang J. A hybrid MCDM model with Monte Carlo simulation to improve decision-making stability and reliability. Inf Sci. (2023) 647:119439. doi: 10.1016/j.ins.2023.119439

[61] Daugavietis JE Soloha R Dace E Ziemele J. A comparison of multi-criteria decision analysis methods for sustainability assessment of district heating systems. Energies. (2022) 15:2411. doi: 10.3390/en15072411

[62] Carpitella S Certa A Izquierdo J la Fata CM. A combined multi-criteria approach to support FMECA analyses: a real-world case. Reliabil Eng Syst Safety. (2018) 169:394–402. doi: 10.1016/j.ress.2017.09.017

[63] Haseli G Deveci M Isik M Gokasar I Pamucar D Hajiaghaei-Keshteli M. Providing climate change resilient land-use transport projects with green finance using Z extended numbers based decision-making model. Expert Syst Appl. (2024) 243:122858. doi: 10.1016/j.eswa.2023.122858

[64] Dhalmahapatra K Garg A Singh K Xavier NF Maiti J. An integrated RFUCOM – RTOPSIS approach for failure modes and effects analysis: a case of manufacturing industry. Reliabil Eng Syst Safety. (2022) 221:108333. doi: 10.1016/j.ress.2022.108333

[65] Ahmed U Carpitella S Certa A. An integrated methodological approach for optimising complex systems subjected to predictive maintenance. Reliabil Eng Syst Safety. (2021) 216:108022. doi: 10.1016/j.ress.2021.108022

[66] Wu Z Tu J. Managing transitivity and consistency of preferences in AHP group decision making based on minimum modifications. Inform Fusion. (2021) 67:125–35. doi: 10.1016/j.inffus.2020.10.012

[67] Rosić M Pešić D Kukić D Antić B Božović M. Method for selection of optimal road safety composite index with examples from DEA and TOPSIS method. Accid Anal Prev. (2017) 98:277–86. doi: 10.1016/j.aap.2016.10.007, PMID: 27792946

[68] Tešić MŽ. Road safety assessment based on a road safety performance index Belgrade: University of Belgrade (2018). 237 p.

[69] World Health Organization. Global status report on road safety: time for action. Geneva, Switzerland: World Health Organization (WHO) (2009).

[70] World Health Organization. Global status report on road safety 2013: supporting a decade of action: summary. Geneva: World Health Organization (2013).

[71] World Health Organization. Global status report on road safety 2015. Geneva: World Health Organization (2015).

[72] World Bank Group, Data: indicators. (2019). Available at: https://data.worldbank.org/indicator

[73] ASEAN Secretariat. ASEAN statistics division (ASEANstats) (2019) (Accessed July 22, 2019); Available at: https://data.aseanstats.org/.

[74] UNDP. Human development report In: United Nations development Programme (UNDP). Washington, DC: Communications Development Incorporated. (2009)

[75] UNDP. Human Development Report 2013 In: United Nations development Programme (UNDP). Washington, DC: Communications Development Incorporated. (2013)

[76] UNDP. Human Development Report 2015 In: United Nations development Programme (UNDP). Washington, DC: Communications Development Incorporated. (2015)

[77] UNDP. Human Development Report 2020 In: United Nations development Programme (UNDP). Washington, DC: Communications Development Incorporated. (2020)

[78] Shannon CE. A mathematical theory of communication. Bell Syst Tech J. (1948) 27:379–423. doi: 10.1002/j.1538-7305.1948.tb01338.x

[79] Brans JP Vincke P Mareschal B. How to select and how to rank projects: the Promethee method. Eur J Oper Res. (1986) 24:228–38. doi: 10.1016/0377-2217(86)90044-5

[80] Ma D Song XB Zhu J Ma W. Input data selection for daily traffic flow forecasting through contextual mining and intra-day pattern recognition. Expert Syst Appl. (2021) 176:114902. doi: 10.1016/j.eswa.2021.114902

[81] Hu L Liu H Zhang J Liu A. KR-DBSCAN: a density-based clustering algorithm based on reverse nearest neighbor and influence space. Expert Syst Appl. (2021) 186:115763. doi: 10.1016/j.eswa.2021.115763

[82] Oladeji I Makolo P Zamora R Lie TT. Density-based clustering and probabilistic classification for integrated transmission-distribution network security state prediction. Electr Power Syst Res. (2022) 211:108164. doi: 10.1016/j.epsr.2022.108164

[83] Campello RJGB Moulavi D Zimek A Sander J. A framework for semi-supervised and unsupervised optimal extraction of clusters from hierarchies. Data Min Knowl Disc. (2013) 27:344–71. doi: 10.1007/s10618-013-0311-4

[84] Campello RJGB Moulavi D Zimek A Sander J. Hierarchical density estimates for data clustering, visualization, and outlier detection. ACM Transact Knowled Discov Data. (2015) 10:1–51. doi: 10.1145/2733381

[85] Dinh D-T Fujinami T Huynh V-N. Estimating the optimal number of clusters in categorical data clustering by Silhouette coefficient In: Knowledge and systems sciences. eds. Chen J, Huynh V, Nguyen GN, Tang X. Singapore: Springer Singapore (2019).

[86] Reddy CK Vinzamuri B. A survey of partitional and hierarchical clustering algorithms In: Data clustering: Chapman and Hall/CRC (2018). 87–110.

[87] Rousseeuw PJ. Silhouettes: a graphical aid to the interpretation and validation of cluster analysis. J Comput Appl Math. (1987) 20:53–65. doi: 10.1016/0377-0427(87)90125-7

[88] Bagirov AM Aliguliyev RM Sultanova N. Finding compact and well-separated clusters: clustering using silhouette coefficients. Pattern Recogn. (2023) 135:109144. doi: 10.1016/j.patcog.2022.109144

[89] Henderi H Wahyuningsih T Rahwanto E. Comparison of min-max normalization and Z-score normalization in the K-nearest neighbor (kNN) algorithm to test the accuracy of types of breast Cancer. Int J Inform Inform Syst. (2021) 4:13–20. doi: 10.47738/ijiis.v4i1.73

[90] Singh D Singh B. Investigating the impact of data normalization on classification performance. Appl Soft Comput. (2020) 97:105524. doi: 10.1016/j.asoc.2019.105524

[91] Mukhametzyanov I. Specific character of objective methods for determining weights of criteria in MCDM problems: entropy, CRITIC and SD. Dec Making. (2021) 4:76–105. doi: 10.31181/dmame210402076i

[92] Chen F Wu J Chen X Wang J Wang D. Benchmarking road safety performance: identifying a meaningful reference (best-in-class). Accid Anal Prev. (2016) 86:76–89. doi: 10.1016/j.aap.2015.10.018 26536072

[93] Fancello G Carta M Fadda P. Road intersections ranking for road safety improvement: comparative analysis of multi-criteria decision making methods. Transp Policy. (2019) 80:188–96. doi: 10.1016/j.tranpol.2018.04.007

[94] Göçer A Özpeynirci Ö Semiz M. Logistics performance index-driven policy development: an application to Turkey. Transp Policy. (2022) 124:20–32. doi: 10.1016/j.tranpol.2021.03.007

[95] Rezaei J van Roekel WS Tavasszy L. Measuring the relative importance of the logistics performance index indicators using best worst method. Transp Policy. (2018) 68:158–69. doi: 10.1016/j.tranpol.2018.05.007

[96] Acheampong AO Opoku EEO Dzator J Kufuor NK. Enhancing human development in developing regions: do ICT and transport infrastructure matter? Technol Forecast Soc Chang. (2022) 180:121725. doi: 10.1016/j.techfore.2022.121725

[97] Düzyol SG Daibaşoğlu K Üzümcüoğlu Y. The relationship between HDI values and road traffic fatality rates. Nesne. (2021) 19:2. doi: 10.7816/nesne-09-19-04

[98] Castro-Nuño M Arévalo-Quijada MT. Assessing urban road safety through multidimensional indexes: application of multi-criteria decision making analysis to rank the Spanish provinces. Transp Policy. (2018) 68:118–29. doi: 10.1016/j.tranpol.2018.04.017

[99] Zu J Peng Z Chen F. Overseeing road safety progress using CV-PROMETHEE II-JSS: a case study in the EU context. Expert Syst Appl. (2022) 195:116623. doi: 10.1016/j.eswa.2022.116623

[100] Chen F Lyu J Wang T. Benchmarking road safety development across OECD countries: an empirical analysis for a decade. Accid Anal Prev. (2020) 147:105752. doi: 10.1016/j.aap.2020.105752, PMID: 32961365

